# Supplementary material for: Characterization of histone acylations links chromatin modifications with metabolism
Source: Nat Commun. 2017 Oct 26;8:1141. doi: 10.1038/s41467-017-01384-9 (PMC5656686; doi:10.1038/s41467-017-01384-9)
Supplement: Supplementary file 1 — Supplementary Information [file 41467_2017_1384_MOESM1_ESM.pdf]

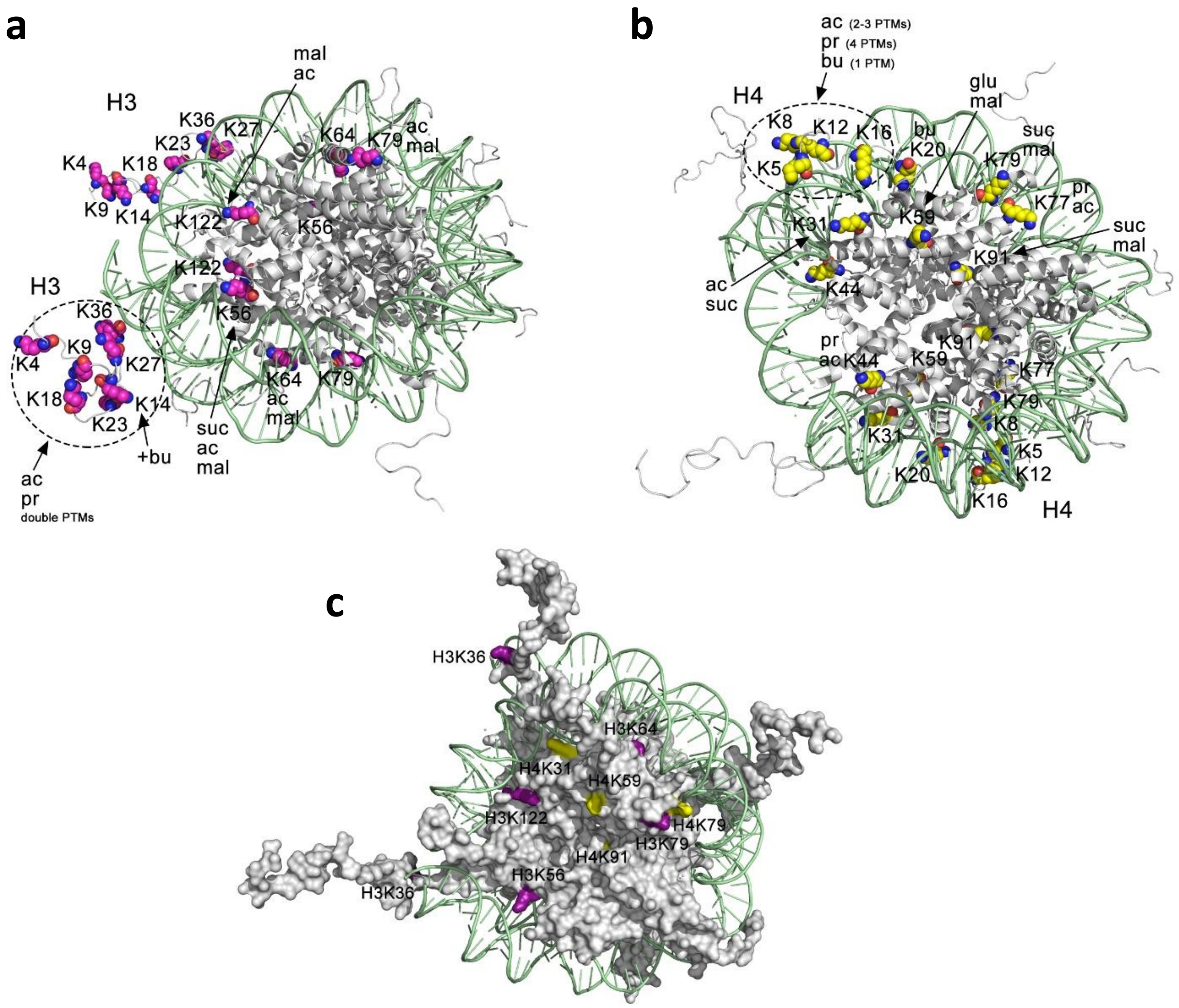

Supplementary Figure 1. Representative nucleosome structure depicting site specificities for enzymatic and non-enzymatic acylations. This figure only represents the mapping of the *in vitro* acylation sites on H3 and H4 if our experiments were carried out using folded histones. Because our experiments were performed in the context of free unfolded histones, this figure is not a realistic representation. (a-b) Crystal structure of the nucleosome (PDB ID: 1KX5) displaying HAT acylation specificities for lysine residues in histone (a) H3 and (B) H4 shown in a space-filling model. Preferential acylation states of each modified lysine are indicated by magenta and yellow colors for histone H3 and H4, respectively. DNA is light green. Lysine modifications are shown as: acetylation (ac), propionylation (pr), butyrylation (bu), crotonylation (cr), malonylation (mal), succinylation (suc), and glutarylation (glu). (c) Most prevalent sites for non-enzymatic acylation are mapped onto the surface representation of the nucleosome structure (PDB ID: 1KX5).

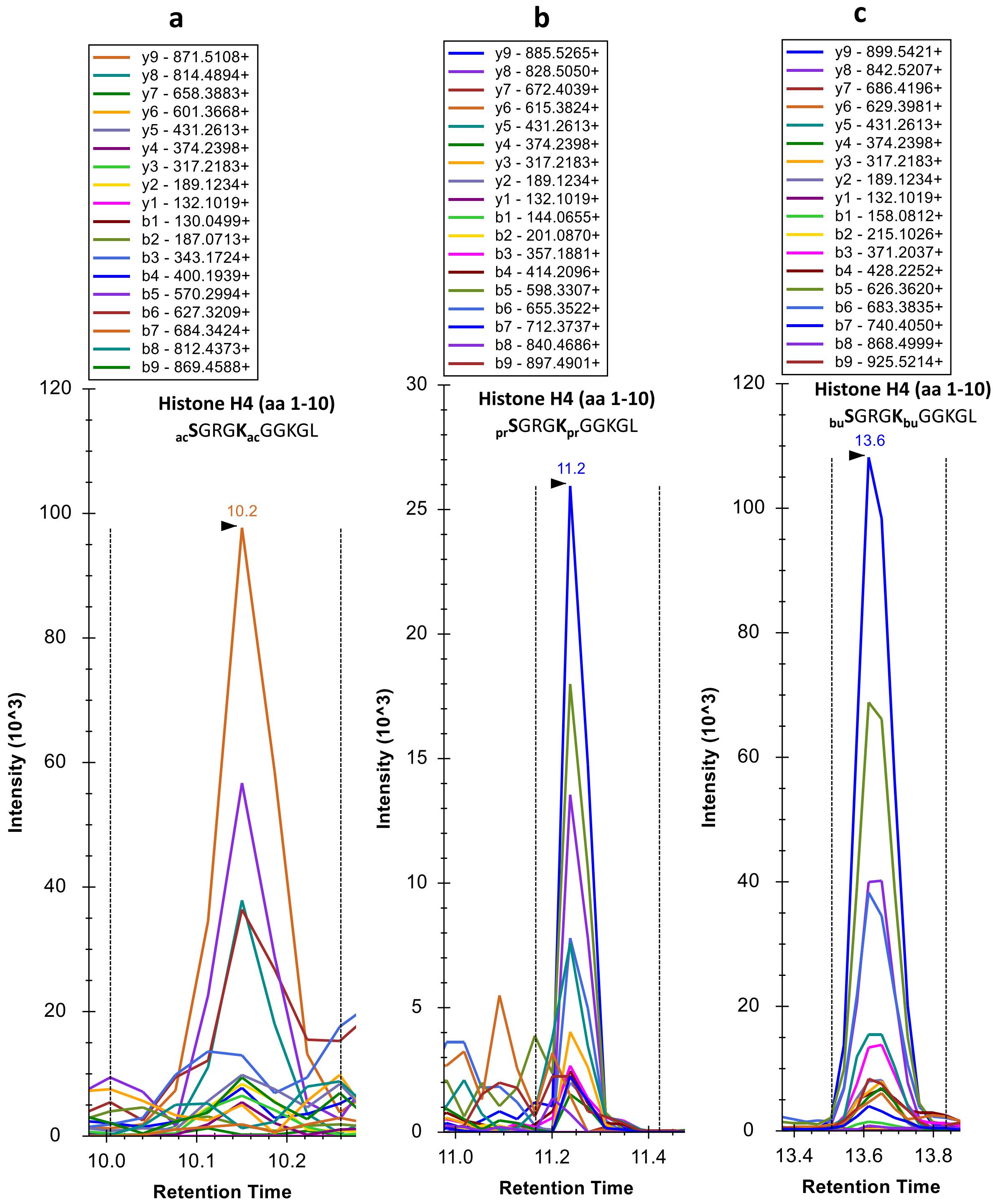

Supplementary Figure 2. Extracted ion chromatograms of N-terminal modifications of histone H4. Panels display product ions of peptide H4 aa 1-10 with N-terminal and K5 (a) acetylation, (b) propionylation, and (c) butyrylation, catalyzed by the N-terminal acetyltransferase NatA *in vitro*. Histone H4 was digested with chymotrypsin to render peptides containing N-terminal serine residue.

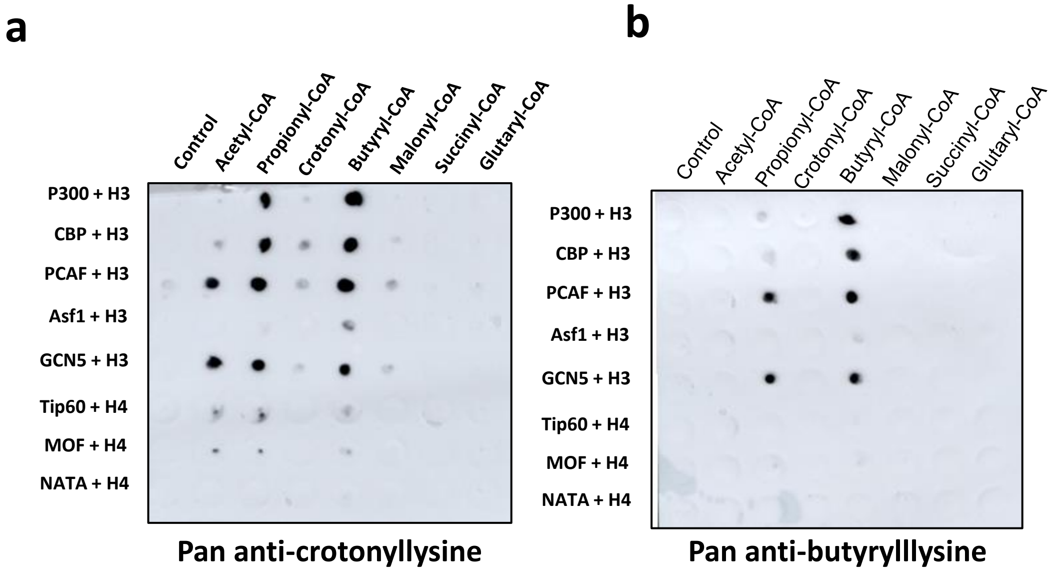

Supplementary Figure 3. Pan acyl-PTM antibody cross-reactivity dot blot analysis. Full length recombinant histones H3 and H4 were incubated with recombinant HATs in the presence of acetyl-, propionyl-, crotonyl-, malonyl-, succinyl-, and glutaryl-CoA. 2μg of enzymatically acylated histones were spotted onto a nitrocellulose membrane and blotted against (a) pan-anticrotonyl-lysine and (b) pan-antibutyryl-lysine. Significant cross-reactivity was observed for pan-anticrotonyl-lysine and pan anti-butyryl-lysine antibodies with structurally closely related acyl marks including lysine acetylation, propionylation, crotonylation and butyrylation.

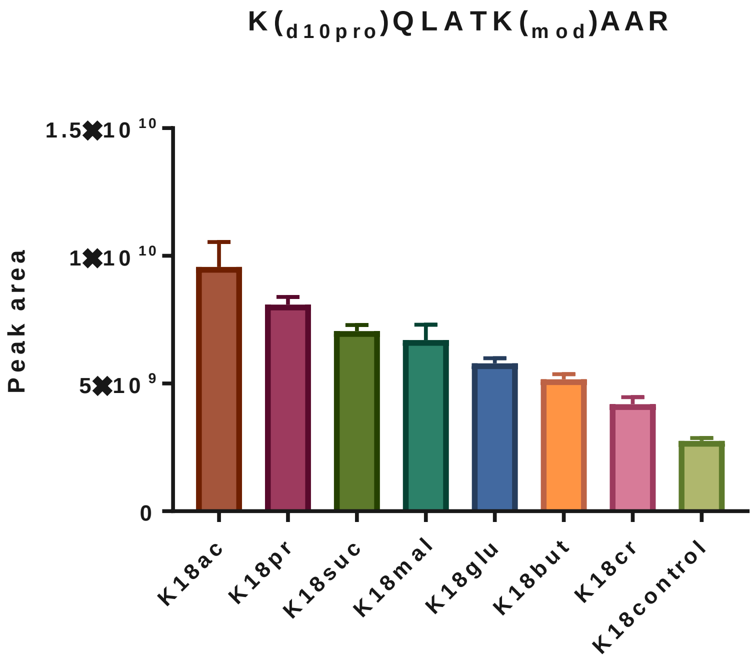

Supplementary Figure 4. Ionization efficiency profiles for differentially acylated peptide H3 aa 18-26 (KQLATKAAR). Bar plots shows the peak areas of synthetic peptide KQLATKAAR with eight different acyl modifications on lysine K18. PTMs (Kac: lysine acetylation, Kcr: lysine crotonylation, Kbu: lysine butyrylation, Kmal: lysine malonylation, Ksu: lysine succinylation, Kglu: lysine glutarylation, and Kpr: lysine propionylation). 2µg of each modified peptide were mixed together, derivatized with d_{10}-propionic anhydride and analyzed by LC-MS. We observed some differences in the ionization efficiency, although all within a maximum of 3-fold changes, but still within a range we consider acceptable. All results are shown as the average of 3 experiments.

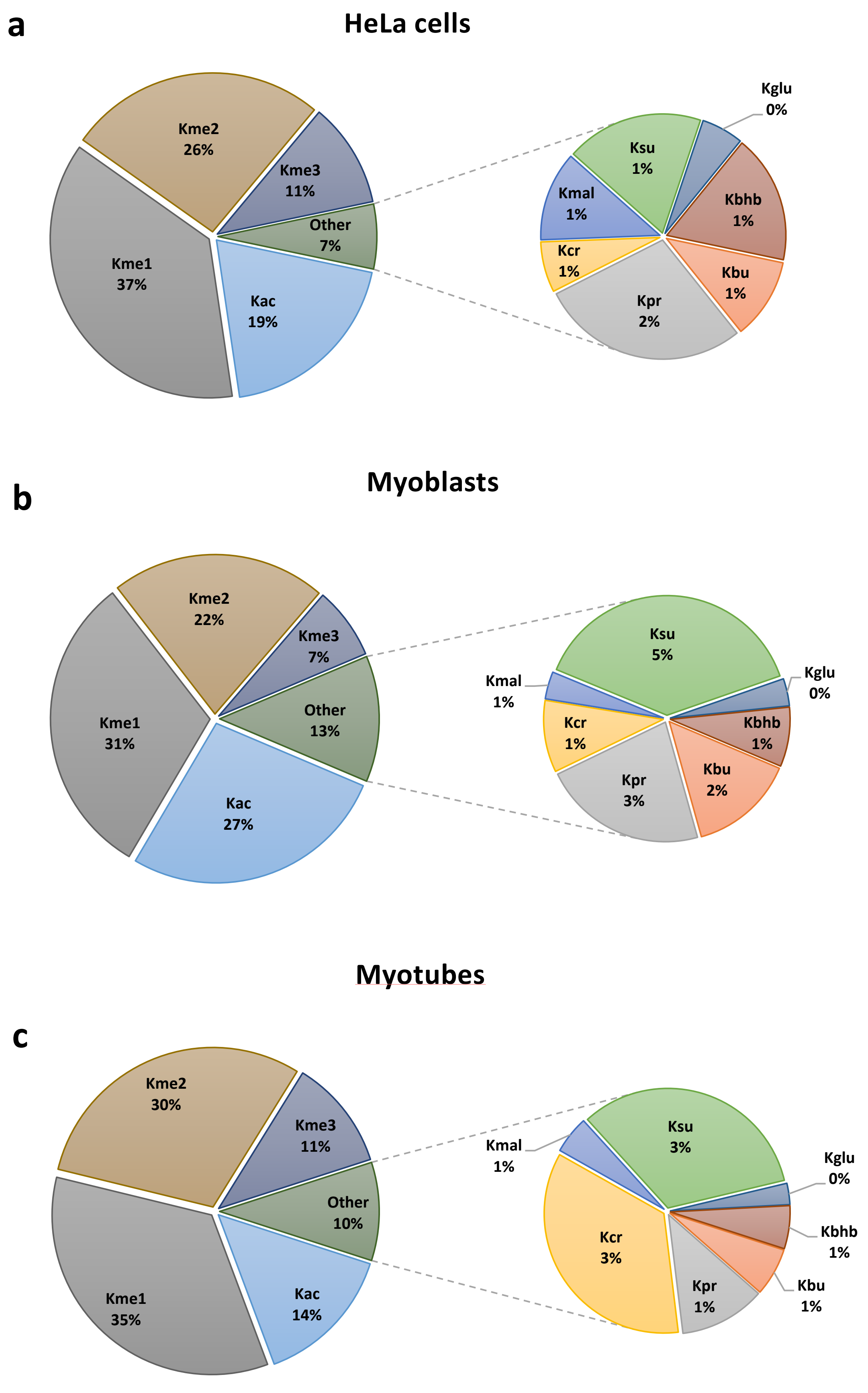

Supplementary figure 5. Overview of the relative abundances of acyl-PTMs on histone H3 and H4. Pie charts showing the relative abundances of 11 lysine PTMs in (A) HeLa cells, (B) Proliferative myogenic cells (myoblasts) and (C) differentiated myogenic cells (myotubes). PTMs (Kac: lysine acetylation, Kme1: lysine monomethylation, Kme2: lysine dimethylation, Kme3: lysine trimethylation, Kcr: lysine crotonylation, Kbu: lysine butyrylation, Kmal: lysine malonylation, Ksu: lysine succinylation, Kglu: lysine glutarylation, and Kpr: lysine propionylation) are shown as percentages representing the sum of the relative abundances of all detectable peptides of canonical histones H3 and H4. All results are shown as the average of 3 biological replicates.

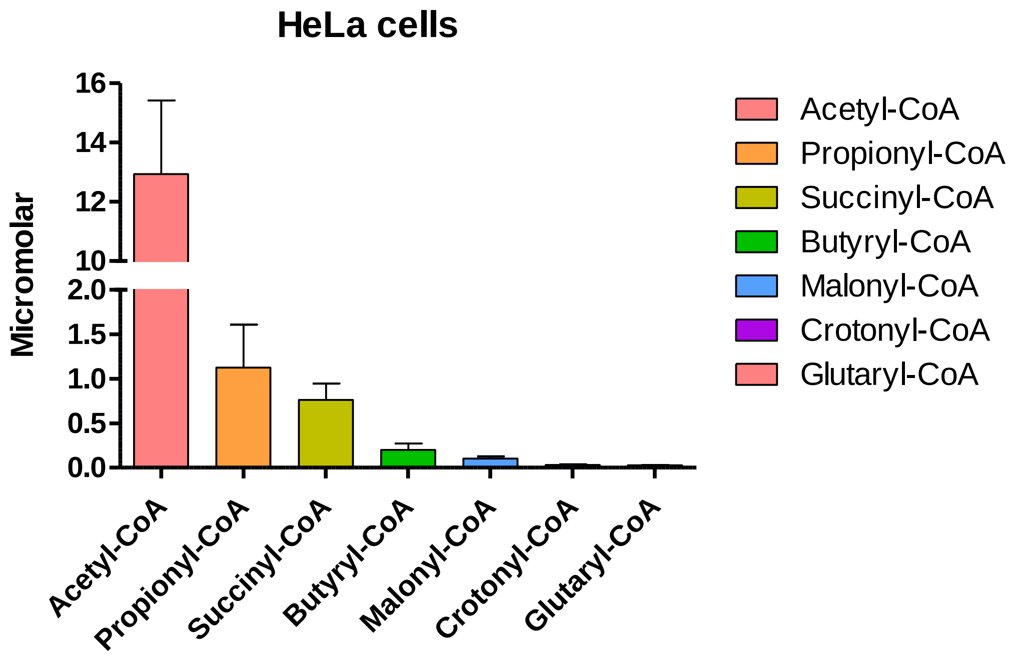

Supplementary Figure 6. Micromolar concentrations of acyl-CoA metabolites in HeLa cells. Bar plots showing the concentrations of acyl-CoA metabolites in HeLa cells normalized to cell volume as determined using a Coulter Z2 Particle Counter.

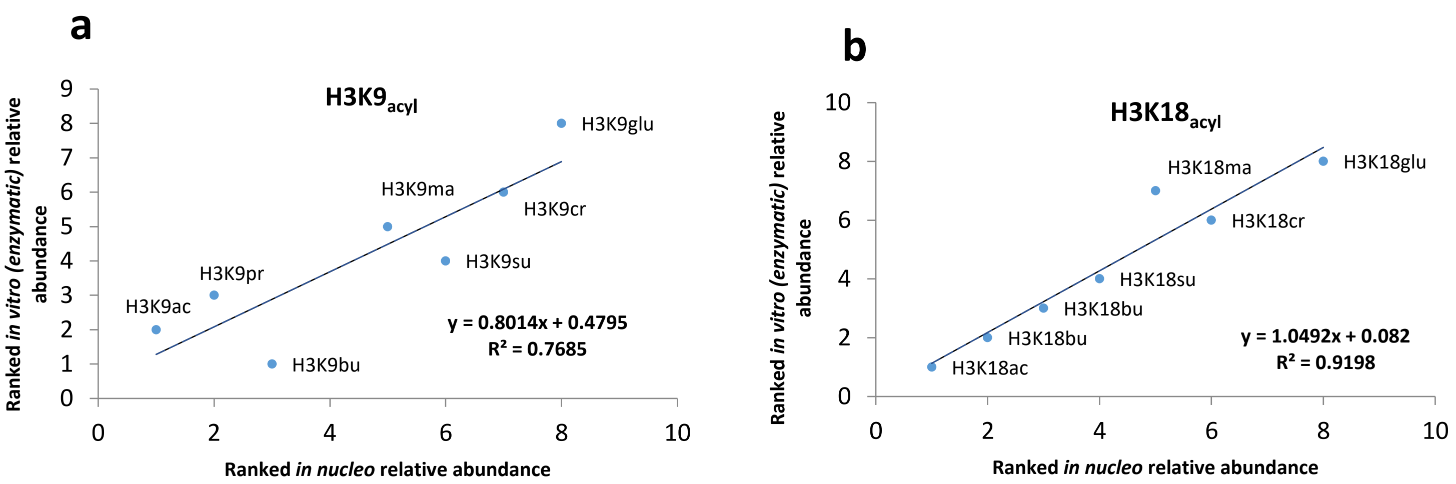

Supplementary figure 7. *In vitro – in nucleo* site occupancy Spearman correlation analysis. Graphs show the correlation between (a) $H3K9_{acyl}$ and (b) $H3K18_{acyl}$ site occupancy in *in vitro* and *in nucleo* experiments. The data represent ranked-order relative abundances of these sites in *in vitro* experiments in the presence of HATs (corrected for non-enzymatic contribution) vs. their abundance in *in nucleo* experiments supplemented with 5 µM acyl-CoAs, K_{bhb} marks were not included in the correlations. Data were displayed as ranks to overcome the issue of the large dynamic range between acetyl marks and all other acyl marks; Pearson correlation would produce high R^2 independently from the relative abundance of the low intensity acyl marks.

Histone H3

Histone H3	CBP							
	Ace	Pro	Cro	But	Mal	Bhb	Suc	Glu
H3_3-8 K4mod	24.1%	4.8%	0.1%	1.6%	0.4%	0.3%	0.8%	0.3%
H3_9-17 K9mod	3.6%	1.2%	0.2%	2.2%	0.5%	0.2%	1.0%	0.1%
H3_9-17 K14mod	34.2%	16.3%	0.3%	5.3%	0.7%	0.2%	1.3%	0.9%
H3_9-17 K9modK14mod	47.7%	11.1%	0.0%	0.5%	0.0%	0.0%	0.0%	0.0%
H38-26 K18mod	16.0%	1.4%	0.2%	4.4%	1.5%	0.5%	1.8%	0.7%
H38-26 K23mod	7.5%	19.7%	0.1%	1.9%	2.6%	0.9%	3.5%	1.7%
H38-26 K18modK23mod	70.5%	12.1%	0.0%	0.3%	0.0%	0.0%	0.0%	0.0%
H3_27-40 K27mod	0.4%	1.2%	0.1%	2.4%	1.5%	0.5%	1.7%	0.8%
H3_27-40 K36mod	7.4%	16.7%	0.1%	3.7%	5.3%	1.6%	3.7%	2.1%
H3_27-40 K27modK36mod	0.3%	4.2%	0.0%	0.1%	0.1%	0.0%	0.0%	0.0%
H3_54-63 K56mod	11.8%	2.7%	0.0%	1.1%	8.2%	1.3%	3.4%	0.7%
H3_64-69 K64mod	5.8%	1.8%	0.0%	2.0%	3.2%	1.2%	2.6%	1.0%
H3_73-83 K79mod	15.9%	2.3%	0.0%	1.5%	4.2%	3.5%	1.2%	1.3%
H317-128 K122mod	7.5%	2.7%	0.0%	2.8%	7.1%	5.8%	7.9%	1.9%
Sum	252.8%	98.1%	1.2%	29.8%	35.3%	16.0%	28.9%	11.5%

Histone H3	GCN5							
	Ace	Pro	Cro	But	Mal	Bhb	Suc	Glu
H3_3-8 K4mod	0.6%	0.9%	0.1%	1.7%	0.3%	0.3%	0.9%	0.4%
H3_9-17 K9mod	0.3%	0.8%	0.3%	0.7%	1.2%	0.2%	1.0%	0.1%
H3_9-17 K14mod	32.2%	11.1%	2.3%	77.2%	3.9%	0.2%	1.4%	1.3%
H3_9-17 K9modK14mod	66.6%	88.1%	0.3%	20.0%	0.0%	0.0%	0.0%	0.0%
H38-26 K18mod	57.8%	3.0%	0.1%	4.8%	1.0%	0.5%	2.1%	0.8%
H38-26 K23mod	1.7%	40.8%	0.1%	13.0%	2.2%	0.9%	4.1%	2.0%
H38-26 K18modK23mod	23.9%	5.5%	0.0%	0.7%	0.0%	0.0%	0.0%	0.0%
H3_27-40 K27mod	6.0%	2.2%	0.1%	6.3%	1.1%	0.5%	1.5%	1.2%
H3_27-40 K36mod	17.7%	29.5%	0.2%	6.0%	3.6%	1.5%	4.1%	2.2%
H3_27-40 K27modK36mod	52.6%	6.0%	0.0%	1.0%	0.0%	0.0%	0.1%	0.0%
H3_54-63 K56mod	3.0%	2.2%	0.0%	1.1%	3.7%	0.8%	4.4%	0.9%
H3_64-69 K64mod	2.6%	0.8%	0.0%	1.5%	3.5%	1.1%	2.8%	1.2%
H3_73-83 K79mod	14.2%	1.9%	0.0%	2.4%	2.8%	3.3%	7.2%	0.8%
H317-128 K122mod	8.4%	1.7%	0.2%	5.4%	6.3%	5.9%	5.6%	2.6%
Sum	287.6%	194.4%	3.7%	141.9%	29.8%	15.2%	35.2%	13.3%

Histone H3	p300							
	Ace	Pro	Cro	But	Mal	Bhb	Suc	Glu
H3_3-8 K4mod	4.5%	5.4%	0.2%	4.9%	0.5%	0.5%	0.7%	0.2%
H3_9-17 K9mod	1.6%	1.1%	0.6%	5.1%	0.6%	0.5%	1.0%	0.1%
H3_9-17 K14mod	12.4%	14.3%	1.8%	6.8%	0.7%	0.6%	1.2%	1.0%
H3_9-17 K9modK14mod	2.2%	10.7%	0.5%	6.4%	0.0%	0.4%	0.0%	0.0%
H38-26 K18mod	18.1%	1.2%	4.1%	9.3%	1.2%	4.0%	2.1%	0.6%
H38-26 K23mod	7.5%	16.1%	0.6%	1.5%	2.6%	2.1%	3.8%	1.6%
H38-26 K18modK23mod	4.1%	11.2%	1.1%	5.1%	0.0%	0.8%	0.0%	0.0%
H3_27-40 K27mod	7.4%	1.1%	1.3%	7.9%	1.4%	1.5%	1.7%	0.9%
H3_27-40 K36mod	8.5%	15.9%	0.4%	4.7%	5.2%	1.9%	2.9%	2.3%
H3_27-40 K27modK36mod	7.3%	5.0%	0.1%	5.0%	0.1%	0.1%	0.1%	0.0%
H3_54-63 K56mod	8.8%	2.7%	0.0%	2.6%	4.6%	1.4%	5.3%	4.7%
H3_64-69 K64mod	4.7%	2.0%	0.0%	1.3%	3.2%	1.1%	2.2%	1.4%
H3_73-83 K79mod	10.6%	2.1%	0.0%	1.0%	6.4%	3.2%	1.8%	0.9%
H317-128 K122mod	10.9%	2.8%	0.2%	3.8%	9.2%	9.1%	2.2%	1.7%
Sum	108.6%	91.6%	11.0%	65.5%	35.8%	27.1%	24.9%	15.4%

Histone H3	pCAF							
	Ace	Pro	Cro	But	Mal	Bhb	Suc	Glu
H3_3-8 K4mod	1.2%	0.6%	0.4%	0.6%	0.4%	0.2%	1.0%	0.2%
H3_9-17 K9mod	0.1%	0.1%	0.3%	1.1%	0.4%	0.2%	1.0%	0.1%
H3_9-17 K14mod	1.9%	1.0%	4.6%	87.6%	0.2%	0.2%	1.2%	1.3%
H3_9-17 K9modK14mod	97.3%	98.9%	0.1%	1.9%	0.0%	0.0%	0.0%	0.0%
H38-26 K18mod	0.9%	4.8%	0.1%	2.4%	1.0%	0.3%	2.2%	0.8%
H38-26 K23mod	25.1%	65.1%	0.1%	3.1%	1.9%	0.5%	3.9%	2.2%
H38-26 K18modK23mod	72.3%	24.0%	0.0%	0.1%	0.0%	0.0%	0.0%	0.0%
H3_27-40 K27mod	1.6%	3.2%	0.1%	2.0%	1.2%	0.3%	1.9%	1.2%
H3_27-40 K36mod	3.2%	43.4%	0.0%	4.6%	3.1%	1.0%	3.6%	2.8%
H3_27-40 K27modK36mod	94.2%	38.4%	0.0%	0.2%	0.0%	0.0%	0.0%	0.0%
H3_54-63 K56mod	2.4%	1.3%	0.0%	0.4%	3.9%	0.3%	3.8%	3.3%
H3_64-69 K64mod	2.9%	1.0%	0.0%	1.4%	3.3%	0.7%	3.0%	1.8%
H3_73-83 K79mod	7.3%	0.8%	0.0%	1.5%	3.9%	0.5%	4.8%	1.9%
H317-128 K122mod	6.3%	0.9%	0.2%	2.8%	0.0%	1.7%	4.8%	4.0%
Sum	316.8%	283.3%	6.0%	109.8%	19.2%	5.9%	31.3%	19.7%

Histone H3	Non-enzymatic							
	Ace	Pro	Cro	But	Mal	Bhb	Suc	Glu
H3_3-8 K4mod	0.5%	1.1%	0.0%	0.9%	0.6%	0.3%	0.9%	0.4%
H3_9-17 K9mod	0.1%	0.2%	0.1%	1.4%	0.7%	0.2%	1.2%	0.1%
H3_9-17 K14mod	0.5%	2.1%	0.0%	1.8%	0.9%	0.2%	1.5%	1.2%
H3_9-17 K9modK14mod	0.0%	0.0%	0.2%	0.0%	0.0%	0.0%	0.0%	0.0%
H38-26 K18mod	0.1%	0.1%	0.0%	2.2%	1.2%	0.5%	2.1%	0.9%
H38-26 K23mod	0.3%	2.1%	0.1%	1.9%	2.0%	0.9%	4.1%	2.0%
H38-26 K18modK23mod	0.0%	0.0%	0.0%	0.2%	0.0%	0.0%	0.0%	0.0%
H3_27-40 K27mod	0.0%	0.5%	0.1%	2.4%	1.4%	0.4%	1.8%	1.4%
H3_27-40 K36mod	4.4%	6.6%	0.1%	2.7%	5.5%	1.7%	3.9%	2.6%
H3_27-40 K27modK36mod	0.1%	0.1%	0.0%	0.1%	0.1%	0.0%	0.0%	0.0%
H3_54-63 K56mod	2.6%	2.6%	0.0%	1.0%	5.4%	1.4%	3.2%	2.9%
H3_64-69 K64mod	2.6%	0.7%	0.0%	2.0%	3.8%	1.5%	2.7%	2.1%
H3_73-83 K79mod	1.6%	1.6%	0.0%	1.5%	3.9%	3.1%	7.8%	2.8%
H317-128 K122mod	4.2%	2.0%	0.0%	2.7%	6.1%	17.1%	4.9%	3.8%
Sum	17.1%	19.8%	0.6%	20.7%	31.6%	27.3%	34.1%	20.3%

Histone H4

Histone H4	NatA							
	Ace	Pro	Cro	But	Mal	Bhb	Suc	Glu
H4_4-17 K5mod	1.3%	2.5%	0.1%	2.8%	2.1%	1.2%	2.9%	2.3%
H4_4-17 K8mod	2.0%	3.3%	0.0%	1.6%	2.9%	0.8%	1.8%	1.1%
H4_4-17 K12mod	0.8%	1.1%	0.1%	3.5%	2.5%	2.2%	2.1%	2.1%
H4_4-17 K16mod	2.1%	2.9%	0.0%	3.7%	3.9%	2.2%	1.9%	3.3%
H4_4-17 2mod	0.5%	0.4%	0.0%	0.3%	0.3%	0.0%	0.3%	0.2%
H4_4-17 3mod	0.0%	0.0%	0.0%	0.1%	0.0%	0.0%	0.0%	0.0%
H4_4-17 4mod	0.0%	0.0%	0.0%	0.0%	0.0%	0.0%	0.0%	0.0%
H4_20-23 K20mod	1.9%	2.8%	0.0%	3.1%	1.1%	1.3%	1.9%	1.5%
H4_24-35 K31mod	5.3%	2.2%	0.1%	3.5%	4.0%	2.4%	3.0%	3.1%
H4_41-45 K44mod	0.0%	0.0%	0.0%	0.0%	0.0%	0.0%	0.0%	0.0%
H4_56-67 K59mod	2.8%	0.0%	0.0%	0.0%	7.7%	1.1%	9.3%	14.2%
H4_68-78 K77mod	1.3%	1.5%	0.0%	0.9%	0.8%	0.6%	1.1%	0.7%
H4_79-92 K79mod	10.0%	2.5%	0.0%	1.2%	8.8%	10.6%	5.6%	3.2%

Histone H4	Tip60							
	Ace	Pro	Cro	But	Mal	Bhb	Suc	Glu
H4_4-17 K5mod	2.5%	2.7%	0.1%	4.0%	2.1%	1.0%	3.2%	2.3%
H4_4-17 K8mod	2.8%	3.2%	0.1%	1.9%	2.6%	0.8%	2.2%	1.1%
H4_4-17 K12mod	1.4%	1.5%	0.1%	4.9%	2.4%	1.8%	2.5%	2.1%
H4_4-17 K16mod	2.5%	3.0%	0.1%	3.9%	3.7%	1.8%	2.9%	3.1%
H4_4-17 2mod	1.5%	0.8%	0.0%	0.7%	0.2%	0.2%	0.3%	0.2%
H4_4-17 3mod	0.2%	0.5%	0.0%	0.4%	0.0%	0.0%	0.0%	0.0%
H4_4-17 4mod	0.2%	0.7%	0.0%	0.1%	0.0%	0.0%	0.0%	0.0%
H4_20-23 K20mod	2.2%	2.6%	0.0%	3.4%	1.7%	1.5%	2.3%	1.4%
H4_24-35 K31mod	4.6%	1.8%	0.2%	3.3%	3.2%	2.0%	2.8%	3.2%
H4_41-45 K44mod	3.6%	0.0%	0.0%	0.0%	0.0%	0.0%	0.0%	0.0%
H4_56-67 K59mod	6.4%	0.0%	0.0%	0.2%	7.3%	6.3%	5.7%	0.3%
H4_68-78 K77mod	1.8%	1.8%	0.0%	1.3%	1.0%	0.8%	1.4%	0.6%
H4_79-92 K79mod	5.4%	2.2%	0.1%	3.3%	9.5%	4.8%	5.5%	3.9%

Histone H4	hMOF							
	Ace	Pro	Cro	But	Mal	Bhb	Suc	Glu
H4_4-17 K5mod	2.2%	0.4%	0.4%	6.7%	2.3%	1.5%	2.8%	2.3%
H4_4-17 K8mod	2.3%	0.1%	0.3%	4.8%	3.0%	1.0%	2.4%	1.1%
H4_4-17 K12mod	0.6%	0.0%	0.3%	6.5%	2.5%	2.1%	2.4%	1.9%
H4_4-17 K16mod	2.6%	0.4%	0.5%	7.3%	4.2%	2.6%	2.7%	3.5%
H4_4-17 2mod	17.5%	2.2%	0.1%	2.9%	0.2%	0.1%	0.3%	0.2%
H4_4-17 3mod	26.1%	13.4%	0.0%	0.4%	0.0%	0.0%	0.0%	0.0%
H4_4-17 4mod	38.6%	78.1%	0.0%	0.1%	0.0%	0.0%	0.0%	0.0%
H4_20-23 K20mod	1.9%	1.1%	0.0%	3.3%	1.7%	0.9%	2.1%	1.4%
H4_24-35 K31mod	11.4%	4.0%	0.3%	3.8%	5.1%	2.2%	3.7%	3.4%
H4_41-45 K44mod	1.3%	0.0%	0.0%	0.0%	0.0%	0.0%	0.0%	0.0%
H4_56-67 K59mod	4.3%	0.0%	0.0%	0.2%	7.2%	0.0%	4.4%	1.4%
H4_68-78 K77mod	19.6%	21.3%	0.0%	1.1%	0.7%	0.5%	1.3%	0.5%
H4_79-92 K79mod	4.0%	0.8%	0.2%	1.9%	8.9%	0.9%	5.8%	4.5%

Histone H4	Non-enzymatic							
	Ace	Pro	Cro	But	Mal	Bhb	Suc	Glu
H4_4-17 K5mod	1.5%	2.8%	0.1%	0.8%	2.4%	1.5%	1.1%	2.1%
H4_4-17 K8mod	2.4%	3.2%	0.0%	0.4%	3.4%	0.9%	0.9%	1.4%
H4_4-17 K12mod	0.9%	1.6%	0.1%	1.0%	2.9%	2.4%	0.9%	2.0%
H4_4-17 K16mod	2.5%	3.0%	0.0%	0.7%	4.6%	2.3%	2.3%	3.6%
H4_4-17 2mod	1.6%	0.4%	0.1%	0.0%	0.5%	0.1%	0.2%	0.3%
H4_4-17 3mod	0.0%	0.0%	0.0%	0.0%	0.0%	0.0%	0.0%	0.0%
H4_4-17 4mod	0.0%	0.0%	0.0%	0.0%	0.0%	0.0%	0.0%	0.0%
H4_20-23 K20mod	2.1%	2.9%	0.0%	0.5%	2.2%	1.3%	1.1%	1.6%
H4_24-35 K31mod	6.8%	2.5%	0.1%	0.1%	5.5%	3.2%	2.7%	2.2%
H4_41-45 K44mod	0.0%	0.0%	0.0%	0.0%	0.0%	0.0%	0.0%	0.0%
H4_56-67 K59mod	8.3%	0.0%	0.0%	0.0%	3.5%	0.0%	3.1%	0.7%
H4_68-78 K77mod	1.7%	1.9%	0.0%	0.1%	0.9%	0.5%	0.6%	0.7%
H4_79-92 K79mod	4.2%	2.3%	0.1%	0.7%	5.8%	10.0%	4.1%	4.8%

**H4_79-92 K91mod**	5.9%	2.0%	0.0%	1.7%	5.9%	8.6%	2.1%	4.6%	3.6%	1.7%	0.1%	3.8%	6.7%	6.5%	9.0%	3.3%	3.4%	0.8%	0.2%	2.4%	6.2%	1.2%	7.7%	3.9%	2.8%	1.7%	0.1%	0.7%	6.1%	9.3%	5.1%	8.0%
**Sum**	33.9%	21.4%	0.4%	22.3%	40.1%	31.0%	31.9%	36.4%	38.6%	22.4%	0.8%	31.1%	40.3%	27.4%	37.8%	21.7%	135.7%	122.7%	2.2%	41.2%	41.9%	13.0%	35.6%	23.9%	34.7%	22.4%	0.6%	5.2%	38.0%	31.5%	21.9%	27.2%

Supplementary Table 1. *In vitro* HAT acylation site specificity. Data represents the relative abundances of the acylated peptides assuming the sum of the raw intensities of the unmodified and modified peptides as 100%. All results are shown as the average of 3 biological replicates.

Peptide

Histone H3

**TKQTAR(H3_3_8)**	**HeLa**	**Myoblasts**	**Myotubes**
H3_3_8 unmod	78.5%	75.2%	78.9%
H3_3_8 K4me1	21.0%	24.0%	21.0%
H3_3_8 K4me2	0.0%	0.0%	0.0%
H3_3_8 K4me3	0.0%	0.0%	0.0%
H3_3_8 K4ac	0.0%	0.1%	0.0%
H3_3_8 K4pr	0.1%	0.1%	0.0%
H3_3_8 K4cr	0.1%	0.0%	0.0%
H3_3_8 K4bu	0.1%	0.1%	0.0%
H3_3_8 K4mal	0.2%	0.0%	0.0%
H3_3_8 K4bhb	0.0%	0.0%	0.0%
H3_3_8 K4suc	0.0%	0.0%	0.0%
H3_3_8 K4glu	0.0%	0.4%	0.0%
**KSTGGKAPR(H3_9_17)**			
H3_9_17 unmod	23.5%	22.3%	23.7%
H3_9_17 K9me1	22.4%	17.7%	27.3%
H3_9_17 K9me2	14.9%	11.0%	17.4%
H3_9_17 K9me3	8.5%	16.3%	12.9%
H3_9_17 K9me1K14ac	8.1%	5.2%	4.4%
H3_9_17 K9me2K14ac	4.9%	4.8%	3.8%
H3_9_17 K9me3K14ac	1.7%	2.7%	1.9%
H3_9_17 K9ac	0.4%	1.9%	0.7%
H3_9_17 K14ac	6.6%	6.5%	4.7%
H3_9_17 K9acK14ac	0.3%	0.4%	0.3%
H3_9_17 K9pr	1.8%	8.2%	0.4%
H3_9_17 K14pr	6.8%	2.1%	1.3%
H3_9_17 K9prK14pr	0.0%	0.1%	0.0%
H3_9_17 K9cr	0.0%	0.0%	0.4%
H3_9_17 K14cr	0.0%	0.0%	0.5%
H3_9_17 K9crK14cr	0.0%	0.0%	0.2%
H3_9_17 K9bu	0.0%	0.0%	0.0%
H3_9_17 K14bu	0.0%	0.0%	0.0%
H3_9_17 K9buK14bu	0.0%	0.0%	0.0%
H3_9_17 K9mal	0.0%	0.0%	0.0%
H3_9_17 K14mal	0.0%	0.0%	0.0%
H3_9_17 K9malK14mal	0.0%	0.0%	0.0%
H3_9_17 K9bhb	0.0%	0.0%	0.0%
H3_9_17 K14bhb	0.0%	0.0%	0.0%
H3_9_17 K9bhbK14bhb	0.0%	0.0%	0.0%
H3_9_17 K9suc	0.0%	0.0%	0.0%

H3_9_17 K14suc	0.0%	0.0%	0.0%
H3_9_17 K9sucK14suc	0.0%	0.0%	0.0%
H3_9_17 K9glu	0.0%	0.0%	0.0%
H3_9_17 K14glu	0.0%	0.0%	0.0%
H3_9_17 K9gluK14glu	0.0%	0.0%	0.0%
**KQLATKAAR(H3_18_26)**			
H3_18_26 unmod	45.6%	69.1%	92.1%
H3_18_26 K23me1	0.2%	0.1%	0.2%
H3_18_26 K18me1	0.2%	0.1%	0.2%
H3_18_26 K18me1K23me1	0.0%	0.0%	0.0%
H3_18_26 K18ac	32.3%	17.1%	2.3%
H3_18_26 K23ac	20.3%	12.2%	4.2%
H3_18_26 K18acK23ac	1.1%	1.1%	0.8%
H3_18_26 K18pr	0.0%	0.0%	0.0%
H3_18_26 K23pr	0.0%	0.0%	0.0%
H3_18_26 K18prK23pr	0.0%	0.0%	0.0%
H3_18_26 K18cr	0.0%	0.0%	0.0%
H3_18_26 K23cr	0.0%	0.0%	0.0%
H3_18_26 K18crK23cr	0.0%	0.0%	0.0%
H3_18_26 K18bu	0.2%	0.1%	0.0%
H3_18_26 K23bu	0.1%	0.0%	0.0%
H3_18_26 K18buK23bu	0.0%	0.0%	0.0%
H3_18_26 K18mal	0.0%	0.0%	0.0%
H3_18_26 K23mal	0.0%	0.0%	0.0%
H3_18_26 K18malK23mal	0.0%	0.0%	0.0%
H3_18_26 K18bhb	0.0%	0.0%	0.0%
H3_18_26 K23bhb	0.0%	0.0%	0.0%
H3_18_26 K18bhbK23bhb	0.0%	0.0%	0.0%
H3_18_26 K18suc	0.0%	0.0%	0.0%
H3_18_26 K23suc	0.0%	0.0%	0.0%
H3_18_26 K18sucK23suc	0.0%	0.0%	0.0%
H3_18_26 K18glu	0.0%	0.0%	0.0%
H3_18_26 K23glu	0.0%	0.0%	0.0%
H3_18_26 K18gluK23glu	0.0%	0.0%	0.0%
**KSAPATGGVKKPHR(H3_27_40)**			
H3_27_40 unmod	6.0%	15.0%	8.1%
H3_27_40 K36me1	1.3%	2.6%	6.2%
H3_27_40 K27me1	5.8%	5.4%	3.0%
H3_27_40 K27me2	23.5%	17.6%	30.3%
H3_27_40 K36me2	4.5%	3.7%	0.9%
H3_27_40 K27me3	11.3%	7.8%	13.9%
H3_27_40 K36me3	2.7%	3.7%	15.7%
H3_27_40 K27me2K36me1	8.6%	9.9%	4.2%

H3_27_40 K27me1K36me2	7.0%	10.1%	5.0%
H3_27_40 K27me1K36me1	1.5%	3.1%	0.3%
H3_27_40 K27me3K36me1	10.4%	5.8%	4.6%
H3_27_40 K27me1K36me3	2.4%	2.9%	1.1%
H3_27_40 K27me2K36me2	12.5%	8.1%	4.7%
H3_27_40 K27me3K36me2	2.1%	3.9%	1.9%
H3_27_40 K27ac	0.1%	0.1%	0.0%
H3_27_40 K36ac	0.1%	0.1%	0.0%
H3_27_40 K27acK36ac	0.0%	0.0%	0.0%
H3_27_40 K27pr	0.0%	0.0%	0.0%
H3_27_40 K36pr	0.0%	0.1%	0.0%
H3_27_40 K27prK36pr	0.0%	0.0%	0.0%
H3_27_40 K27cr	0.0%	0.0%	0.0%
H3_27_40 K36cr	0.0%	0.1%	0.1%
H3_27_40 K27crK36cr	0.0%	0.0%	0.0%
H3_27_40 K27bu	0.0%	0.0%	0.0%
H3_27_40 K36bu	0.0%	0.0%	0.0%
H3_27_40 K27buK36bu	0.0%	0.0%	0.0%
H3_27_40 K27mal	0.0%	0.0%	0.0%
H3_27_40 K36mal	0.0%	0.0%	0.0%
H3_27_40 K27malK36mal	0.0%	0.0%	0.0%
H3_27_40 K27bhb	0.0%	0.0%	0.0%
H3_27_40 K36bhb	0.0%	0.0%	0.0%
H3_27_40 K27bhbK36bhb	0.0%	0.0%	0.0%
H3_27_40 K27suc	0.0%	0.0%	0.0%
H3_27_40 K36suc	0.0%	0.0%	0.0%
H3_27_40 K27sucK36suc	0.0%	0.0%	0.0%
H3_27_40 K27glu	0.0%	0.0%	0.0%
H3_27_40 K36glu	0.0%	0.0%	0.0%
H3_27_40 K27gluK36glu	0.0%	0.0%	0.0%
**YQKSTELLIR(H3_54_63)**			
H3_54_63 unmod	93.6%	93.9%	90.2%
H3_54_63 K56me1	0.6%	0.1%	0.1%
H3_54_63 K56me2	1.9%	1.3%	3.3%
H3_54_63 K56me3	0.1%	0.0%	0.0%
H3_54_63 K56ac	0.2%	0.5%	0.3%
H3_54_63 K56pr	0.2%	0.0%	0.1%
H3_54_63 K56cr	0.3%	0.2%	0.1%
H3_54_63 K56bu	0.2%	0.4%	0.4%
H3_54_63 K56mal	1.6%	0.8%	0.7%
H3_54_63 K56bhb	0.5%	0.1%	0.3%
H3_54_63 K56suc	0.6%	2.6%	2.9%
H3_54_63 K56glu	0.1%	0.1%	0.4%

**KLPFQR(H3_64_69)**			
H3_64_69 unmod	93.7%	93.8%	95.2%
H3_64_69 K64ac	0.8%	0.0%	0.0%
H3_64_69 K64pr	0.0%	0.0%	0.0%
H3_64_69 K64cr	0.0%	0.0%	0.0%
H3_64_69 K64bu	0.0%	0.0%	0.0%
H3_64_69 K64mal	0.1%	0.0%	0.0%
H3_64_69 K64bhb	0.0%	0.0%	0.0%
H3_64_69 K64suc	4.2%	5.9%	4.7%
H3_64_69 K64glu	1.1%	0.2%	0.0%
**EIAQDFKTDLR(H3_73_83)**			
H3_73_83 unmod	26.3%	69.9%	41.6%
H3_73_83 K79me1	2.3%	4.5%	6.7%
H3_73_83 K79me2	70.7%	22.0%	46.3%
H3_73_83 K79me3	0.3%	0.2%	0.0%
H3_73_83 K79ac	0.2%	0.5%	0.1%
H3_73_83 K79pr	0.0%	0.1%	0.0%
H3_73_83 K79cr	0.0%	0.5%	3.9%
H3_73_83 K79bu	0.0%	0.1%	0.1%
H3_73_83 K79mal	0.0%	0.0%	0.0%
H3_73_83 K79bhb	0.0%	0.4%	0.9%
H3_73_83 K79suc	0.0%	1.3%	0.5%
H3_73_83 K79glu	0.0%	0.5%	0.0%
**VTIMPKDIQLAR(H3_117_128)**			
H3_117_128 unmod	77.0%	89.5%	95.0%
H3_117_128 K122me1	5.4%	0.1%	0.2%
H3_117_128 K122me2	0.1%	0.1%	0.0%
H3_117_128 K122me3	7.6%	0.3%	0.4%
H3_117_128 K122ac	3.8%	2.7%	0.2%
H3_117_128 K122pr	0.2%	0.3%	0.5%
H3_117_128 K122cr	2.1%	1.2%	1.2%
H3_117_128 K122bu	2.2%	2.7%	0.2%
H3_117_128 K122mal	0.1%	0.2%	0.0%
H3_117_128 K122bhb	0.2%	0.1%	0.0%
H3_117_128 K122suc	1.1%	2.7%	2.2%
H3_117_128 K122glu	0.2%	0.1%	0.1%

Histone H4

GKGGKGLGKGGAKR(H4_4_17)

H4_4_17 unmod	67.5%	57.8%	72.3%
H4_4_17 K5ac	3.2%	1.5%	1.2%
H4_4_17 K8ac	2.6%	1.9%	2.1%
H4_4_17 K12ac	6.5%	3.5%	2.7%
H4_4_17 K16ac	17.0%	24.5%	14.5%
H4_4_17 K5acK8ac	0.1%	0.4%	0.4%
H4_4_17 K5acK12ac	0.2%	0.6%	0.3%
H4_4_17 K5acK16ac	0.2%	0.6%	0.5%
H4_4_17 K8acK12ac	0.2%	0.3%	0.1%
H4_4_17 K8acK16ac	0.7%	2.6%	1.9%
H4_4_17 K12acK16ac	0.9%	3.7%	2.4%
H4_4_17 K5acK8acK12ac	0.1%	0.2%	0.2%
H4_4_17 K5acK8acK16ac	0.1%	0.3%	0.1%
H4_4_17 K5acK12acK16ac	0.1%	0.3%	0.1%
H4_4_17 K8acK12acK16ac	0.3%	0.9%	0.5%
H4_4_17 K5acK8acK12acK16ac	0.1%	0.3%	0.2%
H4_4_17 K5pr	0.1%	0.1%	0.1%
H4_4_17 K8pr	0.0%	0.0%	0.0%
H4_4_17 K12pr	0.0%	0.0%	0.0%
H4_4_17 K16pr	0.0%	0.0%	0.1%
H4_4_17 2pr	0.0%	0.0%	0.0%
H4_4_17 3pr	0.0%	0.0%	0.0%
H4_4_17 4pr	0.0%	0.0%	0.0%
H4_4_17 K5cr	0.0%	0.0%	0.0%
H4_4_17 K8cr	0.0%	0.0%	0.0%
H4_4_17 K12cr	0.0%	0.0%	0.0%
H4_4_17 K16cr	0.0%	0.0%	0.0%
H4_4_17 2cr	0.0%	0.0%	0.0%
H4_4_17 3cr	0.0%	0.0%	0.0%
H4_4_17 4cr	0.0%	0.0%	0.0%
H4_4_17 K5bu	0.0%	0.0%	0.0%
H4_4_17 K8bu	0.0%	0.0%	0.0%
H4_4_17 K12bu	0.0%	0.0%	0.0%
H4_4_17 K16bu	0.0%	0.0%	0.0%
H4_4_17 2bu	0.0%	0.0%	0.0%
H4_4_17 3bu	0.0%	0.0%	0.0%
H4_4_17 4bu	0.0%	0.0%	0.0%
H4_4_17 K5mal	0.0%	0.0%	0.0%
H4_4_17 K8mal	0.0%	0.0%	0.0%
H4_4_17 K12mal	0.0%	0.0%	0.0%
H4_4_17 K16mal	0.0%	0.0%	0.0%

H4_4_17 2mal	0.0%	0.0%	0.0%
H4_4_17 3mal	0.0%	0.0%	0.0%
H4_4_17 4mal	0.0%	0.0%	0.0%
H4_4_17 K5bhb	0.0%	0.0%	0.0%
H4_4_17 K8bhb	0.0%	0.0%	0.0%
H4_4_17 K12bhb	0.0%	0.0%	0.0%
H4_4_17 K16bhb	0.0%	0.0%	0.0%
H4_4_17 2bhb	0.0%	0.0%	0.0%
H4_4_17 3bhb	0.0%	0.0%	0.0%
H4_4_17 4bhb	0.0%	0.0%	0.0%
H4_4_17 K5suc	0.0%	0.0%	0.0%
H4_4_17 K8suc	0.0%	0.0%	0.0%
H4_4_17 K12suc	0.0%	0.0%	0.0%
H4_4_17 K16suc	0.0%	0.1%	0.1%
H4_4_17 2suc	0.0%	0.0%	0.0%
H4_4_17 3suc	0.0%	0.0%	0.0%
H4_4_17 4suc	0.0%	0.0%	0.0%
H4_4_17 K5glu	0.0%	0.0%	0.0%
H4_4_17 K8glu	0.0%	0.0%	0.0%
H4_4_17 K12glu	0.0%	0.0%	0.0%
H4_4_17 K16glu	0.0%	0.0%	0.0%
H4_4_17 2glu	0.0%	0.0%	0.0%
H4_4_17 3glu	0.0%	0.0%	0.0%
H4_4_17 4glu	0.0%	0.0%	0.0%
**KVLR(H4_20_23)**			
H4_20_23 unmod	24.8%	37.5%	22.1%
H4_20_23 K20me1	49.1%	58.2%	53.8%
H4_20_23 K20me2	1.2%	3.2%	15.3%
H4_20_23 K20me3	24.9%	1.1%	8.8%
H4_20_23 K20ac	0.0%	0.0%	0.0%
H4_20_23 K20pr	0.0%	0.0%	0.0%
H4_20_23 K20cr	0.0%	0.0%	0.0%
H4_20_23 K20bu	0.0%	0.0%	0.0%
H4_20_23 K20mal	0.0%	0.0%	0.0%
H4_20_23 K20bhb	0.0%	0.0%	0.0%
H4_20_23 K20suc	0.0%	0.0%	0.0%
H4_20_23 K20glu	0.0%	0.0%	0.0%
**DNIQGITKPAIR(H4_24_35)**			
H4_24_35 unmod	96.9%	99.3%	99.7%
H4_24_35 K31me1	0.3%	0.1%	0.1%
H4_24_35 K31me2	0.2%	0.1%	0.0%
H4_24_35 K31me3	0.0%	0.0%	0.0%
H4_24_35 K31ac	2.5%	0.0%	0.0%

H4_24_35 K31pr	0.0%	0.4%	0.0%
H4_24_35 K31cr	0.0%	0.1%	0.1%
H4_24_35 K31bu	0.0%	0.0%	0.0%
H4_24_35 K31mal	0.0%	0.0%	0.0%
H4_24_35 K31bhb	0.0%	0.0%	0.0%
H4_24_35 K31suc	0.0%	0.0%	0.0%
H4_24_35 K31glu	0.0%	0.0%	0.0%
**GGVKR(H4_41_45)**			
H4_41_45 unmod	14.9%	97.1%	97.1%
H4_41_45 K44me1	84.0%	1.2%	2.0%
H4_41_45 K44me2	0.7%	0.9%	0.7%
H4_41_45 K44me3	0.0%	0.0%	0.0%
H4_41_45 K44ac	0.0%	0.0%	0.0%
H4_41_45 K44pr	0.0%	0.0%	0.0%
H4_41_45 K44cr	0.0%	0.0%	0.0%
H4_41_45 K44bu	0.1%	0.5%	0.0%
H4_41_45 K44mal	0.0%	0.0%	0.0%
H4_41_45 K44bhb	0.0%	0.0%	0.0%
H4_41_45 K44suc	0.3%	0.1%	0.0%
H4_41_45 K44glu	0.0%	0.2%	0.0%
**GVLKVFLENVIR(H4_56_67)**			
H4_56_67 unmod	98.0%	72.4%	76.9%
H4_68_78 K59me1	0.2%	0.0%	0.3%
H4_68_78 K59me2	0.0%	2.4%	0.0%
H4_68_78 K59me3	0.0%	0.0%	0.0%
H4_56_67 K59ac	0.6%	11.4%	7.9%
H4_56_67 K59pr	0.1%	0.1%	0.5%
H4_56_67 K59cr	0.0%	2.8%	8.5%
H4_56_67 K59bu	0.0%	2.3%	1.2%
H4_56_67 K59mal	0.4%	0.7%	0.5%
H4_56_67 K59bhb	0.0%	1.5%	0.8%
H4_56_67 K59suc	0.5%	6.2%	3.0%
H4_56_67 K59glu	0.3%	0.2%	0.3%
**DAVTYTEHAKR(H4_68_78)**			
H4_68_78 unmod	97.9%	98.4%	98.7%
H4_68_78 K77me1	0.7%	0.2%	0.8%
H4_68_78 K77me2	0.0%	0.0%	0.0%
H4_68_78 K77me3	0.0%	0.0%	0.1%
H4_68_78 K77ac	0.0%	0.0%	0.0%
H4_68_78 K77pr	0.1%	0.0%	0.0%
H4_68_78 K77cr	0.1%	0.0%	0.1%
H4_68_78 K77bu	0.1%	0.1%	0.1%
H4_68_78 K77mal	0.1%	0.0%	0.0%

H4_68_78 K77bhb	0.7%	0.4%	0.1%
H4_68_78 K77suc	0.2%	0.9%	0.1%
H4_68_78 K77glu	0.0%	0.0%	0.0%
**KTVTAMDVVYALKR(H4_79_92)**			
H4_79_92 unmod	92.8%	89.0%	95.6%
H4_79_92 K79ac	0.3%	4.4%	1.7%
H4_79_92 K91ac	0.1%	4.4%	0.6%
H4_79_92 K79pr	0.0%	0.1%	0.1%
H4_79_92 K91pr	0.1%	0.2%	0.1%
H4_79_92 K79cr	0.1%	0.1%	0.2%
H4_79_92 K91cr	0.2%	0.2%	0.2%
H4_79_92 K79bu	0.1%	0.0%	0.0%
H4_79_92 K91bu	1.3%	0.0%	0.0%
H4_79_92 K79mal	0.5%	0.1%	0.1%
H4_79_92 K91mal	1.0%	0.2%	0.4%
H4_79_92 K79bhb	0.9%	0.0%	0.1%
H4_79_92 K91bhb	2.2%	0.1%	0.2%
H4_79_92 K79suc	0.1%	0.6%	0.4%
H4_79_92 K91suc	0.1%	0.3%	0.1%
H4_79_92 K79glu	0.1%	0.0%	0.1%
H4_79_92 K91glu	0.1%	0.3%	0.2%

Supplementary Table 2. ***In vivo*** **acyl-PTMs quantification**. Data represents the relative abundances of the modified peptides assuming the sum of the raw intensities of the unmodified and modified peptides as 100%. All results are shown as the average of 3 biological replicates.

Peptide	GCN5							p300							pCAF							CBP						
**Histone H3**	**ace-pro**	**ace-cro**	**ace-but**	**ace-bhb**	**ace-mal**	**ace-suc**	**ace-glu**	**ace-pro**	**ace-cro**	**ace-but**	**ace-bhb**	**ace-mal**	**ace-suc**	**ace-glu**	**ace-pro**	**ace-cro**	**ace-but**	**ace-bhb**	**ace-mal**	**ace-suc**	**ace-glu**	**ace-pro**	**ace-cro**	**ace-but**	**ace-bhb**	**ace-mal**	**ace-suc**	**ace-glu**
**TKQTAR(H3_3_8)**																												
H3_3_8 K4acetyl	0.78%	0.73%	0.52%	0.65%	0.60%	0.51%	0.51%	3.60%	3.28%	1.89%	3.62%	2.24%	3.16%	2.88%	0.80%	1.29%	1.17%	1.93%	1.06%	1.13%	1.52%	12.18%	18.68%	13.38%	16.27%	30.53%	22.88%	23.48%
H3_3_8 K4acyl	0.46%	0.10%	0.71%	0.08%	0.14%	0.31%	0.35%	1.23%	0.69%	0.93%	0.09%	0.30%	0.36%	0.30%	0.22%	0.32%	0.33%	0.01%	0.07%	0.00%	0.00%	2.67%	0.78%	6.65%	0.14%	0.12%	0.42%	0.24%
**KSTGGKAPR(H3_9_17)**																												
H3_9_17 K9acetyl	5.66%	4.49%	2.09%	0.03%	2.92%	3.26%	2.92%	14.21%	3.84%	3.41%	4.96%	5.87%	3.17%	4.99%	0.01%	0.03%	0.18%	0.25%	0.11%	0.10%	0.09%	5.14%	4.99%	7.47%	11.86%	7.19%	10.18%	10.65%
H3_9_17 K9acyl	0.89%	0.00%	0.14%	0.03%	0.00%	0.00%	0.00%	0.98%	0.21%	2.33%	0.18%	0.30%	0.61%	0.25%	0.03%	0.00%	0.02%	0.07%	0.01%	0.00%	0.00%	5.40%	0.11%	4.98%	0.17%	0.09%	0.11%	0.05%
H3_9_17 K14acetyl	26.42%	20.49%	27.31%	4.44%	16.57%	15.65%	19.96%	19.24%	9.16%	6.79%	12.69%	13.46%	8.70%	11.67%	0.36%	0.14%	0.38%	0.73%	0.27%	0.22%	0.30%	6.38%	14.16%	17.86%	30.26%	17.82%	32.23%	28.82%
H3_9_17 K14acyl	0.00%	0.00%	0.29%	0.03%	0.01%	0.01%	0.00%	1.61%	0.09%	2.95%	0.31%	0.50%	0.77%	0.15%	0.09%	0.00%	0.02%	0.05%	0.01%	0.00%	0.00%	3.73%	0.07%	7.28%	0.60%	0.19%	0.23%	0.08%
H3_9_17 K9acetylK14acetyl	66.91%	69.65%	68.92%	95.30%	79.45%	78.76%	76.76%	2.51%	6.48%	3.41%	5.35%	5.22%	5.94%	4.87%	93.88%	99.51%	98.28%	98.26%	99.40%	98.77%	99.36%	1.96%	28.21%	18.12%	15.04%	53.43%	40.42%	45.62%
H3_9_17 K9acylK14acyl	0.00%	0.21%	0.05%	0.00%	0.00%	0.00%	0.01%	0.00%	0.00%	3.17%	0.00%	0.00%	0.00%	0.00%	5.47%	0.00%	0.04%	0.00%	0.00%	0.00%	0.00%	0.00%	0.00%	4.40%	0.00%	0.00%	0.00%	0.00%
**KQLATKAAR(H3_18_26)**																												
H3_18_26 K18acetyl	3.12%	9.42%	11.17%	6.19%	10.33%	7.88%	7.97%	9.30%	13.43%	9.95%	14.37%	13.91%	12.18%	13.35%	0.81%	2.86%	3.05%	3.35%	2.46%	3.23%	1.36%	8.16%	14.79%	16.29%	30.44%	10.40%	24.05%	21.62%
H3_18_26 K18acyl	0.66%	0.01%	0.87%	0.06%	0.28%	0.71%	0.39%	3.58%	0.08%	9.35%	0.33%	0.90%	1.24%	0.74%	0.31%	0.00%	0.30%	0.00%	0.00%	0.01%	0.00%	8.43%	0.06%	13.53%	1.91%	0.25%	0.27%	0.14%
H3_18_26 K23acetyl	41.32%	39.66%	39.13%	48.24%	45.13%	41.95%	40.21%	7.64%	7.44%	6.17%	8.62%	9.75%	9.41%	9.94%	27.92%	13.94%	24.93%	21.98%	19.36%	12.31%	26.85%	7.13%	8.47%	6.26%	14.30%	7.77%	10.00%	8.67%
H3_18_26 K23acyl	8.45%	0.01%	0.76%	0.16%	0.34%	0.91%	0.77%	0.75%	0.05%	3.19%	0.33%	0.87%	0.74%	0.92%	3.11%	0.00%	0.45%	0.01%	0.01%	0.00%	0.00%	1.78%	0.03%	2.65%	0.68%	0.17%	0.16%	0.08%
H3_18_26 K18acetylK23acetyl	38.35%	10.54%	6.93%	12.13%	7.97%	6.64%	6.86%	9.53%	11.36%	3.13%	8.81%	6.55%	6.72%	6.90%	66.72%	79.07%	69.10%	73.37%	74.71%	83.19%	70.60%	8.60%	29.29%	17.89%	34.29%	61.10%	54.14%	60.52%
H3_18_26 K18acylK23acyl	0.60%	0.00%	0.55%	0.00%	0.00%	0.00%	0.01%	1.07%	0.00%	3.03%	0.00%	0.00%	0.00%	0.00%	0.72%	0.00%	0.02%	0.00%	0.00%	0.00%	0.00%	3.64%	0.00%	7.57%	0.01%	0.00%	0.00%	0.00%
**KSAPATGGVKKPHR(H3_27_40)**																												
H3_27_40 K27acetyl	1.65%	5.35%	9.98%	4.67%	8.14%	2.29%	9.66%	3.31%	2.28%	2.53%	1.96%	5.06%	1.54%	1.87%	0.58%	0.72%	0.26%	1.52%	0.61%	0.21%	0.37%	0.75%	3.27%	10.55%	13.15%	10.41%	5.69%	12.45%
H3_27_40 K27acyl	0.30%	0.03%	0.55%	0.16%	0.25%	0.17%	0.22%	0.21%	0.19%	3.94%	0.31%	0.63%	0.52%	0.30%	0.02%	0.00%	0.00%	0.00%	0.00%	0.00%	0.00%	0.85%	0.00%	1.04%	0.18%	0.12%	0.15%	0.15%
H3_27_40 K36acetyl	39.16%	33.77%	27.38%	39.18%	29.54%	38.28%	27.85%	22.85%	10.12%	7.94%	13.68%	12.16%	10.39%	14.83%	5.23%	5.54%	2.39%	7.03%	3.13%	1.19%	1.49%	12.60%	19.01%	12.99%	25.75%	17.75%	30.18%	31.88%
H3_27_40 K36acyl	3.36%	0.07%	1.93%	0.38%	1.58%	1.08%	1.05%	3.75%	0.14%	5.59%	0.80%	2.32%	1.85%	2.52%	0.22%	1.72%	0.01%	0.00%	0.01%	0.02%	0.00%	6.80%	0.90%	9.35%	0.87%	0.65%	0.84%	0.75%
H3_27_40 K27acetylK36acetyl	42.98%	11.90%	5.68%	9.56%	14.95%	9.48%	11.41%	6.96%	4.07%	0.94%	3.55%	2.58%	3.93%	2.61%	93.06%	86.40%	94.93%	89.67%	89.46%	97.60%	97.53%	4.74%	21.02%	9.11%	15.15%	45.46%	26.87%	19.78%
H3_27_40 K27acylK36acyl	0.45%	0.02%	0.38%	0.00%	0.01%	0.01%	0.00%	0.00%	0.33%	2.77%	0.00%	0.02%	0.01%	0.00%	0.37%	0.00%	0.01%	0.00%	0.00%	0.00%	0.00%	0.71%	0.05%	2.76%	0.00%	0.00%	0.00%	0.00%
**YQKSTELLIR(H3_54_63)**																												
H3_54_63 K56acetyl	1.45%	4.72%	3.87%	4.96%	4.36%	3.45%	3.31%	1.89%	1.35%	2.10%	2.49%	2.60%	2.17%	3.52%	0.71%	1.00%	2.02%	2.00%	1.68%	2.01%	1.69%	1.06%	5.23%	3.92%	4.51%	14.31%	5.94%	7.08%
H3_54_63 K56acyl	1.36%	0.00%	1.59%	0.44%	0.27%	0.81%	0.89%	1.12%	0.00%	1.72%	0.50%	0.51%	0.03%	0.28%	0.76%	0.00%	0.83%	0.27%	0.94%	0.64%	0.51%	1.77%	0.00%	1.28%	0.44%	2.53%	1.03%	1.45%
**KLPFQR(H3_64_69)**																												
H3_64_69 K64acetyl	3.37%	0.61%	1.60%	2.30%	1.37%	2.16%	1.71%	3.45%	1.05%	2.00%	2.96%	2.70%	2.90%	2.03%	2.10%	0.81%	2.05%	3.70%	2.09%	1.84%	1.53%	3.71%	1.84%	2.62%	4.30%	4.19%	2.83%	3.71%
H3_64_69 K64acyl	1.12%	0.05%	1.22%	0.52%	0.78%	0.05%	0.10%	1.04%	0.04%	2.00%	0.65%	0.72%	1.29%	0.95%	0.69%	0.00%	1.25%	0.58%	1.19%	0.75%	0.64%	1.86%	0.03%	1.64%	0.66%	2.12%	1.08%	1.30%

**EIAQDFKTDLR(H3_73_83)**																												
H3_73_83 K79acetyl	3.84%	8.77%	9.17%	4.82%	6.79%	9.01%	3.70%	8.20%	2.88%	21.14%	19.56%	6.24%	4.18%	7.40%	4.98%	2.01%	5.43%	5.05%	1.75%	3.46%	6.60%	7.39%	11.65%	9.48%	12.65%	9.19%	7.80%	6.36%
H3_73_83 K79acyl	0.97%	0.00%	0.00%	1.00%	1.46%	0.35%	0.63%	0.62%	0.00%	0.00%	2.39%	1.85%	0.01%	1.46%	0.44%	0.00%	0.00%	0.86%	0.36%	0.79%	1.22%	1.12%	0.00%	0.00%	1.23%	3.96%	4.61%	3.95%
**VTIMPKDIQLAR(H3_117_128)**																												
H3_117_128 K122acetyl	6.97%	3.01%	5.05%	9.43%	1.39%	6.76%	4.38%	14.24%	10.00%	17.15%	17.04%	32.33%	1.14%	3.33%	2.49%	4.05%	2.41%	4.12%	3.50%	4.73%	3.43%	4.38%	6.34%	2.54%	4.66%	9.86%	3.63%	3.40%
H3_117_128 K122acyl	0.91%	0.00%	2.52%	0.84%	0.54%	0.44%	0.72%	0.67%	0.00%	0.00%	3.38%	1.98%	0.37%	1.22%	0.43%	1.60%	0.04%	0.57%	2.82%	0.54%	1.10%	1.06%	0.00%	0.00%	0.06%	0.79%	0.30%	0.51%

**Histone H4**				**MOF**							**NatA**							**Tip60**			
**GKGGKGLGKGGAKR(H4_4_17)**	**ace-pro**	**ace-cro**	**ace-but**	**ace-bhb**	**ace-mal**	**ace-suc**	**ace-glu**	**ace-pro**	**ace-cro**	**ace-but**	**ace-bhb**	**ace-mal**	**ace-suc**	**ace-glu**	**ace-pro**	**ace-cro**	**ace-but**	**ace-bhb**	**ace-mal**	**ace-suc**	**ace-glu**
H4_4_17 K5acetyl	0.57%	4.14%	4.96%	0.99%	1.61%	1.94%	1.21%	5.73%	0.27%	0.53%	1.23%	0.83%	1.71%	0.96%	3.94%	0.74%	1.19%	1.53%	1.23%	1.58%	0.90%
H4_4_17 K5acyl	0.17%	0.01%	0.21%	0.00%	0.38%	0.02%	0.07%	0.91%	0.05%	1.03%	0.64%	0.02%	0.67%	0.59%	1.18%	0.06%	2.48%	0.49%	1.39%	2.13%	0.78%
H4_4_17 K8acetyl	0.32%	2.44%	1.06%	0.53%	1.05%	1.03%	0.78%	6.93%	0.27%	0.70%	1.21%	0.86%	0.47%	0.45%	5.11%	0.74%	0.83%	1.00%	1.23%	1.14%	0.53%
H4_4_17 K8acyl	0.09%	0.01%	0.06%	0.00%	0.04%	0.00%	0.01%	0.82%	0.04%	0.67%	0.21%	0.05%	0.30%	0.16%	0.76%	0.05%	2.19%	0.18%	1.45%	0.31%	0.33%
H4_4_17 K12acetyl	0.24%	2.63%	0.68%	0.33%	1.03%	1.34%	0.94%	1.48%	0.51%	0.65%	1.61%	0.94%	0.64%	0.87%	3.97%	0.66%	1.46%	1.17%	1.39%	1.61%	1.13%
H4_4_17 K12acyl	0.02%	0.01%	0.04%	0.00%	0.02%	0.00%	0.03%	1.90%	0.03%	1.06%	0.46%	1.46%	0.54%	0.76%	0.94%	0.03%	2.64%	0.25%	1.61%	0.91%	0.90%
H4_4_17 K16acetyl	1.22%	9.56%	2.80%	1.79%	3.69%	3.72%	3.11%	3.06%	1.17%	1.60%	4.61%	2.97%	1.71%	1.65%	2.36%	1.69%	3.01%	3.75%	4.95%	2.95%	2.83%
H4_4_17 K16acyl	0.50%	0.02%	0.17%	0.00%	0.12%	0.01%	0.07%	1.06%	0.06%	1.62%	0.96%	0.94%	0.11%	0.84%	1.12%	0.05%	2.88%	0.76%	3.27%	1.69%	1.97%
H4_4_17 2acetyl	0.81%	17.40%	18.91%	4.94%	11.81%	10.07%	10.11%	0.43%	0.75%	0.06%	0.78%	0.82%	0.18%	0.74%	0.39%	1.03%	0.65%	1.62%	1.86%	0.99%	1.11%
H4_4_17 2acyl	0.70%	0.08%	0.03%	0.00%	0.00%	0.04%	0.00%	0.17%	0.10%	0.19%	0.00%	0.05%	0.05%	0.02%	0.21%	0.13%	0.32%	0.00%	0.07%	0.05%	0.02%
H4_4_17 3acetyl	5.18%	18.62%	23.26%	17.30%	17.78%	15.80%	15.05%	0.02%	0.02%	0.02%	0.96%	0.02%	0.04%	0.53%	0.39%	0.23%	0.18%	1.17%	0.62%	0.31%	0.57%
H4_4_17 3acyl	2.43%	0.07%	0.00%	0.00%	0.00%	0.00%	0.00%	0.00%	0.03%	0.08%	0.00%	0.00%	0.00%	0.00%	0.03%	0.04%	0.07%	0.00%	0.00%	0.00%	0.00%
H4_4_17 4acetyl	63.35%	21.19%	41.36%	68.57%	56.43%	58.23%	57.90%	0.01%	0.01%	0.05%	0.70%	0.03%	0.23%	0.48%	0.24%	0.21%	0.20%	1.47%	0.55%	0.41%	2.99%
H4_4_17 4acyl	14.95%	0.00%	0.00%	0.00%	0.00%	0.00%	0.00%	0.00%	0.00%	0.01%	0.00%	0.00%	0.00%	0.00%	0.02%	0.01%	0.01%	0.00%	0.00%	0.00%	0.00%
**KVLR(H4_20_23)**																					
H4_20_23 K20acetyl	0.48%	0.33%	0.54%	0.46%	0.46%	0.52%	0.74%	3.31%	0.35%	1.13%	1.28%	0.85%	1.01%	0.72%	2.68%	0.38%	0.54%	1.89%	1.42%	0.93%	0.98%
H4_20_23 K20acyl	0.00%	0.06%	1.70%	0.14%	0.87%	0.91%	0.79%	0.00%	0.03%	2.06%	0.30%	0.83%	0.84%	0.65%	0.00%	0.04%	1.55%	0.28%	1.30%	0.74%	0.66%
**DNIQGITKPAIR(H4_24_35)**																					
H4_24_35 K31acetyl	10.88%	4.71%	5.55%	9.05%	9.65%	10.31%	9.66%	7.85%	5.70%	1.66%	3.35%	2.18%	1.00%	3.05%	6.40%	0.91%	1.99%	4.23%	2.68%	3.47%	2.94%
H4_24_35 K31acyl	2.06%	0.99%	2.68%	0.44%	2.43%	2.99%	1.67%	0.71%	0.38%	1.06%	1.48%	2.97%	1.48%	1.77%	0.82%	0.68%	4.78%	0.68%	1.59%	0.73%	0.08%
**GGVKR(H4_41_45)**																					
H4_41_45 K44acetyl	1.50%	1.17%	1.01%	1.79%	1.15%	1.31%	1.58%	0.00%	0.00%	0.00%	0.00%	0.00%	0.00%	0.00%	0.87%	0.00%	0.00%	1.58%	0.70%	0.00%	0.68%
H4_41_45 K44acyl	0.00%	0.00%	0.00%	0.10%	0.00%	0.00%	0.00%	0.00%	0.00%	0.00%	0.00%	0.00%	0.00%	0.00%	0.89%	0.00%	0.00%	0.01%	0.00%	0.00%	0.00%

**GVLKVFLENVIR(H4_56_67)**																					
H4_56_67 K59acetyl	3.96%	3.93%	4.02%	3.54%	11.58%	4.52%	7.35%	5.51%	4.67%	4.66%	12.99%	4.02%	3.16%	5.17%	7.71%	3.29%	9.52%	5.90%	1.03%	2.09%	2.16%
H4_56_67 K59acyl	0.00%	0.00%	0.00%	0.00%	1.33%	1.72%	0.68%	1.68%	0.00%	0.00%	0.00%	1.77%	0.00%	0.00%	0.14%	0.00%	0.00%	0.00%	0.79%	0.73%	0.36%
**DAVTYTEHAKR(H4_68_78)**																					
H4_68_78 K77acetyl	14.16%	5.57%	15.50%	25.51%	24.07%	16.07%	18.71%	1.08%	0.12%	0.44%	1.20%	0.61%	0.36%	6.53%	0.98%	0.32%	0.30%	2.29%	0.56%	0.80%	0.97%
H4_68_78 K77acyl	11.99%	0.00%	1.27%	0.09%	0.32%	0.43%	0.29%	0.76%	0.00%	0.58%	0.10%	0.60%	0.54%	0.41%	1.06%	0.00%	1.52%	0.16%	0.57%	0.83%	0.28%
**KTVTAMDVVYALKR(H4_79_92)**																					
H4_79_92 K79acetyl	3.61%	1.61%	1.51%	1.02%	5.16%	3.52%	5.07%	1.98%	5.54%	8.49%	8.96%	7.21%	4.59%	4.67%	0.63%	0.53%	1.48%	5.63%	4.34%	1.02%	0.18%
H4_79_92 K79acyl	2.67%	0.00%	1.37%	0.20%	1.21%	1.61%	0.35%	0.42%	0.00%	1.04%	0.24%	0.30%	0.79%	0.48%	0.44%	0.00%	0.00%	0.94%	1.93%	1.61%	0.13%
H4_79_92 K91acetyl	1.66%	1.31%	1.35%	1.39%	4.94%	3.12%	5.09%	2.82%	3.92%	7.64%	8.81%	5.04%	4.26%	4.53%	0.23%	0.47%	1.28%	5.72%	4.16%	0.85%	0.13%
H4_79_92 K91acyl	2.74%	0.00%	2.13%	0.24%	0.42%	0.77%	1.14%	0.57%	0.00%	0.09%	0.20%	0.82%	0.72%	0.04%	0.36%	0.00%	0.00%	1.25%	1.54%	0.58%	0.38%

Supplementary Table 3. ***In vitro* HAT competition assay**. Competition assay in the presence of 10 μM acetyl-CoA and 10 μM of a competing acyl-donor. Data represents the relative abundances of the modified peptides assuming the sum of the raw intensities of the unmodified and modified peptides as 100%. All results are shown as the average of 2 biological replicates. All values shown were corrected by the contribution of non-enzymatic acylation.

Peptide	Control	Acetyl-CoA_1uM	Acetyl-CoA_5uM	Propionyl-CoA_1uM	Propionyl-CoA_5uM	Crotonyl-CoA_1uM	Crotonyl-CoA_5uM	Butyryl-CoA_1uM	Butyryl-CoA_5uM	Malonyl-CoA_1uM	Malonyl-CoA_5uM	β-hydroxybutytyl-CoA_1uM	β-hydroxybutytyl-CoA_5uM	Succinyl-CoA_1uM	Succinyl-CoA_5uM	Glutaryl-CoA_1uM	Glutaryl-CoA_5uM
TKQTAR(H3_3_8)																	
H3_3_8 unmod	91.0%	87.01%	89.32%	78.58%	87.01%	90.73%	84.26%	71.45%	94.67%	86.55%	84.70%	82.97%	89.30%	88.24%	89.73%	87.61%	88.68%
H3_3_8 K4me1	8.75%	12.79%	10.10%	17.65%	10.01%	9.11%	15.49%	27.80%	3.88%	13.09%	14.83%	16.22%	10.53%	11.54%	9.89%	11.76%	10.71%
H3_3_8 K4me2	0.04%	0.01%	0.01%	0.00%	0.01%	0.01%	0.04%	0.00%	0.00%	0.04%	0.04%	0.02%	0.02%	0.03%	0.02%	0.08%	0.02%
H3_3_8 K4me3	0.01%	0.00%	0.06%	0.00%	0.00%	0.04%	0.00%	0.09%	0.00%	0.01%	0.25%	0.08%	0.03%	0.02%	0.22%	0.27%	0.11%
H3_3_8 K4ac	0.03%	0.02%	0.02%	0.00%	0.00%	0.01%	0.01%	0.08%	0.01%	0.01%	0.00%	0.00%	0.02%	0.02%	0.03%	0.03%	0.02%
H3_3_8 K4gl	0.05%	0.00%	0.02%	0.00%	0.00%	0.01%	0.01%	0.03%	0.01%	0.01%	0.00%	0.00%	0.01%	0.04%	0.00%	0.01%	0.01%
H3_3_8 K4hi	0.03%	0.02%	0.01%	0.00%	0.02%	0.03%	0.07%	0.36%	0.54%	0.10%	0.06%	0.20%	0.03%	0.06%	0.04%	0.15%	0.06%
H3_3_8 K4ma	0.00%	0.02%	0.01%	0.00%	0.00%	0.01%	0.00%	0.04%	0.06%	0.00%	0.02%	0.07%	0.00%	0.01%	0.02%	0.01%	0.06%
H3_3_8 K4su	0.00%	0.08%	0.00%	0.00%	0.00%	0.00%	0.11%	0.13%	0.33%	0.05%	0.00%	0.00%	0.01%	0.00%	0.02%	0.00%	0.03%
H3_3_8 K4cr	0.00%	0.03%	0.01%	3.52%	2.78%	0.02%	0.01%	0.02%	0.06%	0.01%	0.00%	0.01%	0.02%	0.01%	0.01%	0.01%	0.00%
H3_3_8 K4bu	0.01%	0.02%	0.01%	0.04%	0.06%	0.00%	0.01%	0.00%	0.43%	0.03%	0.05%	0.03%	0.00%	0.00%	0.00%	0.03%	0.04%
H3_3_8 K4pr	0.00%	0.02%	0.43%	0.20%	0.10%	0.03%	0.00%	0.00%	0.00%	0.10%	0.04%	0.40%	0.02%	0.04%	0.01%	0.04%	0.27%
KSTGGKAPR(H3_9_17)																	
H3_9_17 unmod	33.9%	33.51%	28.70%	27.13%	32.88%	36.69%	33.43%	27.53%	30.00%	35.71%	36.11%	24.03%	34.82%	28.14%	30.97%	31.69%	47.38%
H3_9_17 K9me1	30.5%	32.76%	28.67%	29.15%	28.72%	25.41%	34.42%	46.96%	59.83%	28.38%	28.76%	38.56%	30.98%	33.50%	28.58%	29.89%	40.55%
H3_9_17 K9me2	16.4%	14.15%	20.99%	19.00%	10.72%	19.58%	13.48%	12.09%	0.02%	16.14%	12.99%	18.37%	16.18%	18.13%	18.48%	17.80%	0.13%
H3_9_17 K9me3	10.0%	10.48%	11.74%	11.42%	12.91%	11.90%	10.31%	7.31%	0.01%	10.12%	9.39%	12.19%	9.53%	11.78%	12.79%	11.33%	0.03%
H3_9_17 K9ac	0.24%	0.39%	0.58%	0.37%	0.66%	0.83%	1.11%	0.61%	0.00%	1.33%	0.85%	0.36%	1.44%	1.28%	0.52%	0.24%	1.02%
H3_9_17 K14ac	3.94%	4.10%	5.23%	2.80%	1.69%	3.54%	3.09%	3.82%	0.00%	3.40%	5.80%	3.30%	2.54%	2.97%	3.79%	4.72%	4.95%
H3_9_17 K9gl	0.00%	0.00%	0.01%	0.00%	0.00%	0.01%	0.00%	0.00%	0.00%	0.01%	0.00%	0.01%	0.00%	0.00%	0.01%	0.00%	0.00%
H3_9_17 K14gl	0.01%	0.00%	0.03%	0.00%	0.00%	0.01%	0.00%	0.00%	0.00%	0.01%	0.00%	0.03%	0.00%	0.00%	0.01%	0.01%	0.00%
H3_9_17 K9hi	0.00%	0.00%	0.00%	0.01%	0.00%	0.00%	0.00%	0.03%	0.00%	0.00%	0.00%	0.00%	0.00%	0.00%	0.01%	0.00%	0.01%
H3_9_17 K14hi	0.01%	0.00%	0.00%	0.02%	0.00%	0.01%	0.00%	0.08%	0.00%	0.00%	0.01%	0.00%	0.00%	0.01%	0.00%	0.00%	0.01%
H3_9_17 K9ma	0.01%	0.01%	0.00%	0.01%	0.01%	0.00%	0.01%	0.01%	0.03%	0.00%	0.01%	0.00%	0.01%	0.02%	0.00%	0.01%	0.01%
H3_9_17 K14ma	0.00%	0.00%	0.00%	0.25%	0.15%	0.01%	0.02%	0.02%	0.03%	0.01%	0.04%	0.01%	0.02%	0.09%	0.01%	0.11%	0.01%
H3_9_17 K9su	0.01%	0.00%	0.00%	0.02%	0.00%	0.00%	0.00%	0.02%	0.05%	0.00%	0.00%	0.01%	0.00%	0.00%	0.00%	0.14%	0.02%
H3_9_17 K14su	0.01%	0.00%	0.01%	0.04%	0.00%	0.00%	0.00%	0.04%	0.10%	0.00%	0.00%	0.03%	0.00%	0.00%	0.00%	0.01%	0.03%
H3_9_17 K9cr	0.00%	0.00%	0.00%	0.01%	0.02%	0.01%	0.00%	0.05%	0.08%	0.00%	0.00%	0.01%	0.01%	0.00%	0.28%	0.00%	0.09%
H3_9_17 K14cr	0.00%	0.00%	0.00%	0.02%	0.05%	0.04%	0.00%	0.36%	0.21%	0.00%	0.00%	0.02%	0.04%	0.00%	0.76%	0.00%	0.10%

H3_9_17 K9bu	0.00%	0.01%	0.06%	0.13%	0.27%	0.02%	0.00%	0.00%	0.06%	0.00%	0.01%	0.00%	0.02%	0.01%	0.00%	0.00%	0.01%
H3_9_17 K14bu	0.01%	0.08%	0.05%	0.00%	0.00%	0.01%	0.00%	0.00%	0.15%	0.01%	0.02%	0.00%	0.04%	0.01%	0.00%	0.00%	0.02%
H3_9_17 K9pr	1.34%	0.41%	1.30%	1.68%	1.72%	0.56%	0.51%	0.89%	2.81%	0.51%	1.46%	0.60%	0.66%	0.47%	0.79%	1.06%	1.32%
H3_9_17 K14pr	3.45%	4.08%	2.62%	7.94%	10.21%	1.36%	3.59%	0.18%	6.62%	4.36%	4.55%	2.46%	3.73%	3.60%	3.00%	2.97%	4.31%
KQLATKAAR(H3_18_26)																	
H3_18_26 unmod	88.9%	87.04%	75.51%	73.25%	64.39%	91.53%	83.35%	91.73%	90.50%	88.70%	88.08%	87.47%	86.04%	90.84%	94.20%	89.78%	91.43%
H3_18_26 K23me1	0.09%	0.10%	0.13%	1.93%	1.97%	0.11%	0.05%	0.13%	0.05%	0.06%	0.04%	0.13%	0.09%	0.10%	0.08%	0.16%	0.09%
H3_18_26 K18me1	0.35%	0.29%	0.42%	5.98%	5.91%	0.49%	0.29%	0.29%	0.24%	0.27%	0.33%	0.33%	0.25%	0.34%	0.38%	0.33%	0.41%
H3_18_26 K18ac	1.83%	2.29%	7.77%	1.53%	2.22%	1.26%	4.81%	1.17%	1.09%	1.82%	1.76%	2.28%	3.92%	1.78%	0.78%	1.37%	1.09%
H3_18_26 K23ac	8.40%	9.78%	15.74%	5.44%	8.99%	5.75%	11.00%	5.60%	6.90%	8.47%	9.44%	9.34%	9.15%	6.57%	4.09%	7.54%	6.44%
H3_18_26 K18gl	0.01%	0.00%	0.00%	0.04%	0.10%	0.00%	0.00%	0.00%	0.00%	0.00%	0.00%	0.00%	0.00%	0.00%	0.00%	0.00%	0.00%
H3_18_26 K23gl	0.01%	0.00%	0.00%	0.11%	0.29%	0.00%	0.00%	0.01%	0.00%	0.00%	0.00%	0.00%	0.00%	0.00%	0.00%	0.00%	0.00%
H3_18_26 K18hi	0.01%	0.00%	0.00%	0.04%	0.00%	0.00%	0.01%	0.00%	0.00%	0.03%	0.03%	0.01%	0.00%	0.01%	0.00%	0.00%	0.00%
H3_18_26 K23hi	0.02%	0.01%	0.00%	0.12%	0.00%	0.01%	0.02%	0.00%	0.00%	0.03%	0.04%	0.01%	0.01%	0.00%	0.00%	0.02%	0.00%
H3_18_26 K18ma	0.01%	0.07%	0.02%	1.86%	1.79%	0.14%	0.06%	0.05%	0.15%	0.08%	0.01%	0.08%	0.09%	0.02%	0.01%	0.07%	0.05%
H3_18_26 K23ma	0.11%	0.16%	0.09%	4.33%	3.71%	0.32%	0.18%	0.32%	0.21%	0.28%	0.04%	0.13%	0.23%	0.07%	0.02%	0.43%	0.15%
H3_18_26 K18su	0.00%	0.00%	0.00%	0.00%	0.27%	0.00%	0.00%	0.00%	0.00%	0.00%	0.00%	0.00%	0.00%	0.00%	0.00%	0.00%	0.00%
H3_18_26 K23su	0.00%	0.00%	0.00%	0.00%	0.59%	0.00%	0.00%	0.00%	0.00%	0.00%	0.00%	0.00%	0.00%	0.00%	0.00%	0.00%	0.00%
H3_18_26 K18cr	0.01%	0.00%	0.00%	0.00%	0.00%	0.00%	0.01%	0.01%	0.00%	0.00%	0.00%	0.00%	0.00%	0.00%	0.00%	0.00%	0.00%
H3_18_26 K23cr	0.02%	0.00%	0.00%	0.02%	0.01%	0.01%	0.01%	0.02%	0.00%	0.00%	0.00%	0.00%	0.01%	0.00%	0.00%	0.00%	0.00%
H3_18_26 K18bu	0.05%	0.05%	0.06%	0.45%	0.84%	0.04%	0.05%	0.27%	0.13%	0.03%	0.03%	0.05%	0.03%	0.06%	0.10%	0.05%	0.05%
H3_18_26 K23bu	0.13%	0.10%	0.20%	0.21%	0.21%	0.21%	0.09%	0.24%	0.63%	0.11%	0.12%	0.12%	0.12%	0.13%	0.22%	0.15%	0.17%
H3_18_26 K18pr	0.02%	0.04%	0.02%	1.14%	3.41%	0.04%	0.01%	0.04%	0.02%	0.03%	0.02%	0.02%	0.02%	0.02%	0.03%	0.02%	0.02%
H3_18_26 K23pr	0.04%	0.07%	0.05%	3.55%	5.29%	0.10%	0.07%	0.11%	0.07%	0.09%	0.06%	0.04%	0.04%	0.05%	0.07%	0.06%	0.09%
KSAPATGGVKKPHR(H3_27_40)																	
H3_27_40 unmod	20.0%	19.74%	19.07%	19.59%	19.31%	18.01%	17.16%	18.60%	45.37%	14.92%	12.90%	21.53%	18.56%	19.64%	20.50%	18.85%	17.26%
H3_27_40 K36me1	6.05%	9.52%	1.46%	7.05%	6.93%	5.32%	4.66%	5.79%	14.68%	2.81%	3.52%	2.34%	6.03%	8.17%	9.16%	9.62%	9.26%
H3_27_40 K27me1	20.0%	16.15%	29.77%	23.49%	21.21%	26.59%	13.09%	26.27%	38.94%	11.62%	9.24%	21.12%	17.98%	20.79%	20.53%	18.24%	20.37%
H3_27_40 K27me2	16.9%	16.35%	16.75%	22.31%	24.10%	15.91%	21.11%	15.98%	0.07%	23.58%	25.46%	17.82%	17.52%	15.17%	15.48%	16.00%	16.55%
H3_27_40 K36me2	16.9%	16.35%	16.75%	5.17%	5.31%	15.91%	21.11%	15.98%	0.07%	23.58%	25.46%	17.82%	17.52%	15.17%	15.48%	16.00%	16.55%
H3_27_40 K27me3	9.62%	10.74%	7.84%	8.19%	8.84%	8.92%	11.27%	8.56%	0.03%	11.57%	11.56%	9.45%	10.97%	10.41%	9.26%	9.25%	9.29%
H3_27_40 K36me3	9.62%	10.74%	7.84%	13.83%	13.88%	8.92%	11.27%	8.56%	0.03%	11.57%	11.56%	9.45%	10.97%	10.41%	9.26%	9.25%	9.29%

H3_27_40 K27ac	0.02%	0.05%	0.07%	0.03%	0.03%	0.09%	0.04%	0.05%	0.10%	0.05%	0.05%	0.06%	0.08%	0.03%	0.03%	0.04%	0.08%
H3_27_40 K36ac	0.14%	0.13%	0.10%	0.14%	0.14%	0.05%	0.15%	0.11%	0.57%	0.13%	0.12%	0.10%	0.10%	0.13%	0.10%	0.10%	0.51%
H3_27_40 K27gl	0.00%	0.00%	0.00%	0.00%	0.00%	0.00%	0.01%	0.00%	0.00%	0.00%	0.00%	0.01%	0.00%	0.00%	0.00%	0.00%	0.00%
H3_27_40 K36gl	0.00%	0.00%	0.00%	0.00%	0.02%	0.00%	0.02%	0.01%	0.00%	0.00%	0.01%	0.03%	0.00%	0.00%	0.02%	0.00%	0.00%
H3_27_40 K27hi	0.00%	0.00%	0.00%	0.00%	0.00%	0.00%	0.00%	0.00%	0.00%	0.00%	0.00%	0.00%	0.00%	0.00%	0.00%	0.00%	0.00%
H3_27_40 K36hi	0.00%	0.00%	0.01%	0.01%	0.01%	0.01%	0.01%	0.00%	0.00%	0.01%	0.01%	0.00%	0.01%	0.01%	0.01%	0.00%	0.01%
H3_27_40 K27ma	0.01%	0.00%	0.02%	0.00%	0.01%	0.01%	0.01%	0.00%	0.00%	0.00%	0.01%	0.01%	0.01%	0.00%	0.03%	0.05%	0.03%
H3_27_40 K36ma	0.08%	0.00%	0.23%	0.02%	0.04%	0.21%	0.04%	0.01%	0.00%	0.06%	0.02%	0.05%	0.02%	0.01%	0.08%	0.20%	0.17%
H3_27_40 K27su	0.00%	0.00%	0.00%	0.01%	0.01%	0.00%	0.00%	0.00%	0.00%	0.00%	0.00%	0.00%	0.00%	0.00%	0.00%	0.00%	0.00%
H3_27_40 K36su	0.00%	0.00%	0.00%	0.04%	0.04%	0.00%	0.00%	0.00%	0.00%	0.00%	0.00%	0.00%	0.00%	0.00%	0.00%	0.00%	0.00%
H3_27_40 K27cr	0.20%	0.10%	0.02%	0.01%	0.02%	0.00%	0.00%	0.00%	0.05%	0.00%	0.00%	0.02%	0.04%	0.00%	0.00%	0.07%	0.03%
H3_27_40 K36cr	0.08%	0.02%	0.02%	0.03%	0.02%	0.00%	0.00%	0.00%	0.02%	0.00%	0.00%	0.10%	0.11%	0.00%	0.00%	2.23%	0.03%
H3_27_40 K27bu	0.00%	0.00%	0.00%	0.01%	0.00%	0.02%	0.00%	0.00%	0.02%	0.02%	0.00%	0.00%	0.00%	0.00%	0.00%	0.03%	0.03%
H3_27_40 K36bu	0.01%	0.00%	0.00%	0.02%	0.01%	0.03%	0.00%	0.00%	0.05%	0.08%	0.00%	0.01%	0.00%	0.00%	0.00%	0.04%	0.26%
H3_27_40 K27pr	0.02%	0.03%	0.03%	0.01%	0.02%	0.00%	0.02%	0.03%	0.00%	0.00%	0.02%	0.02%	0.01%	0.03%	0.04%	0.00%	0.07%
H3_27_40 K36pr	0.06%	0.06%	0.05%	0.03%	0.03%	0.00%	0.04%	0.04%	0.00%	0.00%	0.04%	0.05%	0.06%	0.04%	0.03%	0.01%	0.18%
YQKSTELLIR(H3_54_63)																	
H3_54_63 unmod	83.7%	92.04%	93.12%	44.25%	30.35%	84.52%	75.55%	91.52%	79.54%	74.84%	61.27%	87.61%	76.60%	93.04%	90.80%	74.72%	63.77%
H3_54_63 K56me1	1.92%	1.02%	0.39%	1.19%	0.11%	0.59%	0.53%	0.03%	1.56%	1.00%	0.72%	0.06%	0.02%	0.59%	1.07%	3.81%	2.36%
H3_54_63 K56me2	12.2%	4.06%	3.98%	35.20%	54.77%	3.00%	5.02%	1.21%	11.34%	8.20%	10.30%	4.50%	3.80%	2.66%	3.00%	11.11%	20.74%
H3_54_63 K56me3	0.08%	0.16%	0.07%	0.19%	0.20%	0.03%	0.00%	0.02%	0.11%	0.19%	0.07%	0.07%	0.04%	0.07%	0.04%	0.14%	0.00%
H3_54_63 K56ac	0.33%	1.02%	0.51%	2.54%	1.73%	0.67%	2.14%	0.70%	4.21%	0.67%	0.52%	2.57%	0.85%	1.33%	0.75%	4.64%	7.28%
H3_54_63 K56gl	0.36%	0.47%	0.08%	0.16%	0.89%	0.05%	0.60%	0.00%	1.11%	0.15%	0.33%	0.89%	0.37%	0.00%	0.11%	0.12%	0.00%
H3_54_63 K56hi	0.12%	0.50%	0.27%	4.74%	4.43%	7.23%	6.78%	0.77%	0.36%	0.11%	0.08%	3.10%	17.59%	1.22%	0.65%	2.58%	2.46%
H3_54_63 K56ma	1.11%	0.70%	1.17%	11.08%	6.21%	3.84%	9.17%	5.65%	1.45%	14.66%	26.65%	0.95%	0.67%	0.53%	0.64%	2.04%	3.14%
H3_54_63 K56su	0.02%	0.04%	0.35%	0.53%	0.89%	0.07%	0.17%	0.00%	0.23%	0.08%	0.01%	0.20%	0.01%	0.54%	2.88%	0.82%	0.06%
H3_54_63 K56cr	0.00%	0.00%	0.01%	0.00%	0.01%	0.01%	0.02%	0.09%	0.09%	0.03%	0.00%	0.05%	0.04%	0.00%	0.01%	0.00%	0.14%
H3_54_63 K56bu	0.01%	0.00%	0.00%	0.04%	0.14%	0.00%	0.00%	0.00%	0.00%	0.02%	0.05%	0.00%	0.00%	0.00%	0.00%	0.00%	0.00%
H3_54_63 K56pr	0.03%	0.00%	0.05%	0.08%	0.27%	0.00%	0.00%	0.01%	0.00%	0.03%	0.00%	0.01%	0.01%	0.02%	0.04%	0.02%	0.05%
KLPFQR(H3_64_69)																	
H3_64_69 unmod	87.7%	94.31%	96.99%	94.33%	88.31%	96.89%	82.11%	96.91%	70.30%	71.43%	72.17%	90.17%	95.46%	94.83%	97.26%	82.55%	86.86%
H3_64_69 K64me1	0.00%	0.00%	0.00%	0.01%	0.00%	0.08%	0.01%	0.00%	0.00%	0.00%	0.03%	0.00%	0.00%	0.00%	0.00%	0.00%	0.00%

H3_64_69 K64me2	0.14%	0.06%	0.01%	0.03%	0.06%	0.00%	0.23%	0.06%	0.00%	0.16%	0.19%	0.05%	0.01%	0.03%	0.02%	0.15%	0.00%
H3_64_69 K64me3	11.2%	5.14%	2.36%	5.02%	10.71%	2.83%	17.13%	1.52%	28.80%	27.86%	23.00%	9.24%	4.45%	4.96%	2.56%	16.31%	11.92%
H3_64_69 K64ac	0.11%	0.36%	0.54%	0.06%	0.00%	0.00%	0.06%	0.97%	0.03%	0.06%	4.27%	0.00%	0.00%	0.03%	0.03%	0.42%	0.54%
H3_64_69 K64gl	0.00%	0.00%	0.01%	0.08%	0.25%	0.01%	0.00%	0.02%	0.03%	0.00%	0.00%	0.05%	0.02%	0.02%	0.00%	0.05%	0.14%
H3_64_69 K64hi	0.00%	0.00%	0.00%	0.02%	0.00%	0.00%	0.00%	0.02%	0.00%	0.00%	0.01%	0.02%	0.00%	0.00%	0.00%	0.04%	0.01%
H3_64_69 K64ma	0.00%	0.00%	0.00%	0.00%	0.00%	0.04%	0.00%	0.01%	0.00%	0.10%	0.00%	0.00%	0.00%	0.00%	0.00%	0.00%	0.04%
H3_64_69 K64su	0.00%	0.01%	0.00%	0.00%	0.00%	0.00%	0.03%	0.00%	0.00%	0.04%	0.04%	0.00%	0.00%	0.00%	0.00%	0.00%	0.04%
H3_64_69 K64cr	0.21%	0.01%	0.00%	0.00%	0.00%	0.00%	0.00%	0.16%	0.00%	0.00%	0.01%	0.00%	0.00%	0.01%	0.00%	0.00%	0.00%
H3_64_69 K64bu	0.04%	0.00%	0.00%	0.11%	0.14%	0.00%	0.02%	0.00%	0.00%	0.00%	0.02%	0.00%	0.01%	0.00%	0.00%	0.04%	0.05%
H3_64_69 K64pr	0.50%	0.11%	0.09%	0.35%	0.53%	0.15%	0.42%	0.32%	0.84%	0.34%	0.25%	0.47%	0.05%	0.13%	0.13%	0.42%	0.41%
EIAQDFKTDLR(H3_73_83)																	
H3_73_83 unmod	6.33%	3.90%	15.07%	54.51%	54.72%	49.08%	26.60%	36.87%	28.20%	9.69%	4.79%	8.51%	42.39%	6.43%	16.59%	7.61%	4.83%
H3_73_83 K79me1	12.6%	6.61%	2.43%	4.53%	4.39%	3.55%	2.93%	1.40%	0.11%	10.36%	2.41%	1.09%	0.94%	5.28%	13.34%	1.12%	26.50%
H3_73_83 K79me2	75.1%	81.43%	75.28%	40.27%	40.32%	40.39%	61.38%	47.71%	62.28%	71.94%	85.59%	84.31%	55.67%	78.46%	57.41%	82.44%	57.94%
H3_73_83 K79me3	0.00%	1.73%	0.32%	0.00%	0.25%	0.00%	0.27%	1.91%	3.89%	0.28%	1.06%	0.14%	0.11%	0.51%	0.33%	0.44%	5.15%
H3_73_83 K79ac	0.00%	0.09%	0.64%	0.00%	0.00%	0.18%	0.00%	0.03%	0.00%	0.00%	0.00%	0.00%	0.00%	0.00%	0.06%	0.22%	0.00%
H3_73_83 K79gl	0.00%	1.86%	0.23%	0.40%	0.20%	1.64%	2.00%	2.13%	1.07%	3.40%	1.61%	0.48%	0.00%	3.76%	0.00%	4.56%	4.29%
H3_73_83 K79hi	0.01%	0.00%	0.00%	0.01%	0.02%	0.00%	0.42%	0.01%	0.00%	1.36%	2.80%	0.79%	0.13%	0.00%	0.13%	0.39%	0.09%
H3_73_83 K79ma	0.25%	0.65%	0.46%	0.14%	0.05%	0.26%	0.45%	0.55%	0.68%	0.00%	0.37%	1.41%	0.45%	0.00%	1.08%	0.00%	0.00%
H3_73_83 K79su	4.42%	3.21%	5.51%	0.02%	0.00%	4.62%	1.98%	8.87%	3.78%	2.46%	1.37%	2.03%	0.10%	5.56%	11.06%	3.23%	1.19%
H3_73_83 K79cr	0.00%	0.00%	0.07%	0.00%	0.01%	0.22%	2.31%	0.41%	0.00%	0.00%	0.01%	0.00%	0.00%	0.00%	0.00%	0.00%	0.00%
H3_73_83 K79bu	0.02%	0.10%	0.00%	0.00%	0.00%	0.07%	0.00%	0.00%	0.00%	0.00%	0.00%	0.00%	0.00%	0.00%	0.00%	0.00%	0.00%
H3_73_83 K79pr	1.18%	0.41%	0.00%	0.11%	0.04%	0.00%	1.65%	0.11%	0.00%	0.50%	0.00%	1.24%	0.20%	0.00%	0.00%	0.00%	0.00%
VTIMPKDIQLAR(H3_117_128)																	
H3_117_128 unmod	72.0%	55.84%	73.40%	85.86%	77.20%	75.75%	66.75%	50.06%	46.76%	79.18%	81.33%	85.17%	85.62%	85.85%	54.48%	58.42%	28.66%
H3_117_128 K122me1	0.22%	7.21%	0.00%	0.03%	0.03%	0.00%	0.00%	4.06%	0.00%	0.00%	0.00%	0.00%	0.85%	0.00%	0.02%	0.00%	0.00%
H3_117_128 K122me2	12.4%	14.32%	9.06%	8.53%	13.69%	7.64%	9.17%	5.16%	10.87%	7.81%	5.74%	6.58%	3.67%	4.52%	10.47%	23.00%	18.20%
H3_117_128 K122ac	3.91%	5.14%	6.21%	0.59%	1.71%	0.28%	3.09%	0.00%	2.54%	1.06%	0.58%	0.97%	0.83%	0.13%	8.78%	2.81%	18.60%
H3_117_128 K122gl	4.05%	7.16%	6.87%	0.86%	1.24%	7.97%	6.79%	0.49%	4.38%	6.28%	1.18%	3.06%	3.07%	6.04%	10.16%	10.20%	20.46%
H3_117_128 K122hi	0.02%	0.00%	0.00%	0.49%	0.44%	0.40%	0.00%	0.79%	0.54%	1.29%	0.89%	0.63%	0.26%	0.00%	0.00%	0.00%	0.00%
H3_117_128 K122ma	2.99%	1.02%	0.00%	0.02%	0.17%	0.00%	2.25%	2.28%	0.52%	0.00%	0.05%	0.00%	0.00%	0.00%	0.00%	0.00%	0.00%
H3_117_128 K122su	2.43%	4.88%	0.47%	0.84%	2.55%	0.38%	0.33%	3.05%	2.71%	3.38%	2.04%	2.48%	2.64%	1.13%	2.96%	5.13%	4.01%

H3_117_128 K122cr	0.00%	0.22%	1.26%	0.00%	0.03%	0.72%	9.58%	0.00%	1.64%	0.00%	3.92%	0.00%	0.12%	1.16%	0.00%	0.00%	6.33%
H3_117_128 K122bu	0.18%	0.91%	0.20%	1.36%	1.40%	5.20%	1.00%	22.46%	23.77%	0.78%	3.67%	1.09%	2.95%	0.67%	0.20%	0.18%	0.50%
H3_117_128 K122pr	1.70%	3.30%	2.54%	1.42%	1.54%	1.66%	1.03%	11.64%	6.25%	0.23%	0.60%	0.03%	0.00%	0.49%	12.93%	0.25%	3.23%
GKGGKGLGKGGAKR(H4_4_17)																	
H4_4_17 unmod	79.1%	59.44%	50.54%	70.03%	75.43%	69.49%	72.26%	75.32%	71.60%	71.99%	69.54%	80.00%	58.30%	51.94%	77.92%	74.70%	91.95%
H4_4_17 1me1	0.00%	0.02%	0.00%	0.03%	0.00%	0.01%	0.02%	0.00%	0.00%	0.01%	0.00%	0.00%	0.00%	0.02%	0.00%	0.19%	0.00%
H4_4_17 2me1	0.01%	0.06%	0.00%	0.00%	0.00%	0.00%	0.00%	0.01%	0.05%	0.04%	0.00%	0.01%	0.01%	0.02%	0.02%	0.02%	0.00%
H4_4_17 1ac	18.0%	35.26%	38.69%	27.03%	19.64%	8.54%	17.88%	11.30%	14.92%	12.10%	24.67%	14.17%	26.48%	41.46%	18.28%	13.52%	3.02%
H4_4_17 2ac	1.97%	3.30%	5.05%	2.37%	1.85%	6.06%	3.29%	5.49%	11.88%	13.57%	5.44%	2.89%	11.96%	5.38%	1.74%	6.94%	3.44%
H4_4_17 1gl	0.01%	0.03%	0.02%	0.00%	0.00%	0.02%	0.00%	0.04%	0.03%	0.01%	0.00%	0.00%	0.02%	0.00%	0.00%	0.02%	0.01%
H4_4_17 2gl	0.00%	0.00%	0.00%	0.00%	0.00%	0.00%	0.00%	0.00%	0.00%	0.00%	0.00%	0.00%	0.00%	0.00%	0.00%	0.00%	0.00%
H4_4_17 1hi	0.00%	0.00%	0.00%	0.00%	0.00%	0.00%	0.00%	0.00%	0.00%	0.00%	0.00%	0.00%	0.00%	0.00%	0.00%	0.02%	0.01%
H4_4_17 2hi	0.00%	0.00%	0.01%	0.17%	0.22%	1.28%	0.33%	2.62%	0.40%	0.99%	0.15%	0.42%	1.14%	0.31%	0.36%	0.91%	0.05%
H4_4_17 1ma	0.03%	0.01%	0.00%	0.01%	0.00%	0.00%	0.03%	0.03%	0.02%	0.06%	0.02%	0.01%	0.01%	0.00%	0.01%	0.04%	0.00%
H4_4_17 2ma	0.00%	0.00%	0.00%	0.00%	0.00%	0.00%	0.00%	0.00%	0.02%	0.00%	0.01%	0.00%	0.00%	0.00%	0.00%	0.00%	0.00%
H4_4_17 1su	0.00%	0.00%	0.00%	0.01%	0.02%	0.00%	0.00%	0.00%	0.00%	0.00%	0.00%	0.00%	0.00%	0.00%	0.00%	0.00%	0.00%
H4_4_17 2su	0.00%	0.07%	0.00%	0.00%	0.00%	0.00%	0.02%	0.30%	0.06%	0.00%	0.01%	0.62%	0.02%	0.03%	0.02%	0.19%	0.00%
H4_4_17 1cr	0.00%	0.01%	0.00%	0.13%	0.17%	0.00%	0.00%	0.09%	0.00%	0.00%	0.00%	0.00%	0.00%	0.00%	0.00%	0.04%	0.00%
H4_4_17 2cr	0.50%	1.24%	4.32%	0.02%	0.01%	14.01%	5.99%	3.08%	0.60%	0.60%	0.01%	1.34%	1.55%	0.44%	0.64%	2.51%	1.34%
H4_4_17 1bu	0.00%	0.00%	0.05%	0.00%	0.00%	0.00%	0.00%	0.00%	0.02%	0.00%	0.00%	0.00%	0.01%	0.01%	0.00%	0.10%	0.00%
H4_4_17 2bu	0.15%	0.50%	1.21%	0.06%	0.02%	0.47%	0.08%	1.28%	0.29%	0.23%	0.08%	0.45%	0.46%	0.32%	0.43%	0.73%	0.09%
H4_4_17 1pr	0.06%	0.04%	0.03%	0.12%	2.36%	0.12%	0.08%	0.17%	0.08%	0.12%	0.05%	0.08%	0.05%	0.04%	0.09%	0.07%	0.07%
H4_4_17 2pr	0.01%	0.01%	0.06%	0.01%	0.26%	0.00%	0.02%	0.26%	0.04%	0.28%	0.02%	0.00%	0.00%	0.03%	0.48%	0.01%	0.01%
KVLR(H4_20_23)																	
H4_20_23 unmod	100%	100.00%	100.00%	81.03%	77.53%	100.00%	100.00%	100.00%	100.00%	100.00%	100.00%	100.00%	100.00%	100.00%	100.00%	100.00%	100.00%
H4_20_23 K20me1	0.00%	0.00%	0.00%	0.00%	0.00%	0.00%	0.00%	0.00%	0.00%	0.00%	0.00%	0.00%	0.00%	0.00%	0.00%	0.00%	0.00%
H4_20_23 K20me2	0.00%	0.00%	0.00%	14.88%	17.69%	0.00%	0.00%	0.00%	0.00%	0.00%	0.00%	0.00%	0.00%	0.00%	0.00%	0.00%	0.00%
H4_20_23 K20me3	0.00%	0.00%	0.00%	4.06%	4.78%	0.00%	0.00%	0.00%	0.00%	0.00%	0.00%	0.00%	0.00%	0.00%	0.00%	0.00%	0.00%
H4_20_23 K20ac	0.00%	0.00%	0.00%	0.00%	0.00%	0.00%	0.00%	0.00%	0.00%	0.00%	0.00%	0.00%	0.00%	0.00%	0.00%	0.00%	0.00%
H4_20_23 K20gl	0.00%	0.00%	0.00%	0.02%	0.00%	0.00%	0.00%	0.00%	0.00%	0.00%	0.00%	0.00%	0.00%	0.00%	0.00%	0.00%	0.00%
H4_20_23 K20hi	0.00%	0.00%	0.00%	0.00%	0.00%	0.00%	0.00%	0.00%	0.00%	0.00%	0.00%	0.00%	0.00%	0.00%	0.00%	0.00%	0.00%
H4_20_23 K20ma	0.00%	0.00%	0.00%	0.00%	0.00%	0.00%	0.00%	0.00%	0.00%	0.00%	0.00%	0.00%	0.00%	0.00%	0.00%	0.00%	0.00%

H4_20_23 K20su	0.00%	0.00%	0.00%	0.00%	0.00%	0.00%	0.00%	0.00%	0.00%	0.00%	0.00%	0.00%	0.00%	0.00%	0.00%	0.00%	0.00%
H4_20_23 K20cr	0.00%	0.00%	0.00%	0.01%	0.00%	0.00%	0.00%	0.00%	0.00%	0.00%	0.00%	0.00%	0.00%	0.00%	0.00%	0.00%	0.00%
H4_20_23 K20bu	0.00%	0.00%	0.00%	0.00%	0.00%	0.00%	0.00%	0.00%	0.00%	0.00%	0.00%	0.00%	0.00%	0.00%	0.00%	0.00%	0.00%
H4_20_23 K20pr	0.00%	0.00%	0.00%	0.00%	0.00%	0.00%	0.00%	0.00%	0.00%	0.00%	0.00%	0.00%	0.00%	0.00%	0.00%	0.00%	0.00%
DNIQGITKPAIR(H4_24_35)																	
H4_24_35 unmod	96.9%	97.36%	98.37%	99.24%	98.70%	97.72%	97.03%	97.38%	95.12%	97.81%	98.62%	97.99%	98.50%	99.11%	98.49%	95.67%	97.71%
H4_24_35 K31me1	0.15%	0.11%	0.24%	0.06%	0.05%	0.02%	0.07%	0.11%	0.09%	0.04%	0.03%	0.04%	0.02%	0.07%	0.09%	0.43%	0.03%
H4_24_35 K31me2	0.00%	0.00%	0.00%	0.00%	0.00%	0.00%	0.00%	0.00%	0.00%	0.00%	0.00%	0.01%	0.00%	0.00%	0.00%	0.00%	0.00%
H4_24_35 K31me3	0.00%	0.05%	0.00%	0.09%	0.22%	0.00%	0.03%	0.01%	0.03%	0.00%	0.01%	0.01%	0.00%	0.01%	0.00%	0.01%	0.00%
H4_24_35 K31ac	0.46%	0.17%	0.27%	0.01%	0.09%	0.22%	0.54%	0.15%	0.58%	0.33%	0.20%	0.25%	0.20%	0.13%	0.12%	0.37%	0.24%
H4_24_35 K31gl	0.01%	0.00%	0.00%	0.00%	0.01%	0.00%	0.00%	0.01%	0.02%	0.00%	0.01%	0.00%	0.00%	0.00%	0.00%	0.03%	0.01%
H4_24_35 K31hi	0.08%	0.07%	0.20%	0.00%	0.01%	0.27%	0.20%	0.09%	0.19%	0.14%	0.00%	0.41%	0.39%	0.10%	0.06%	0.13%	0.05%
H4_24_35 K31ma	0.00%	0.74%	0.07%	0.25%	0.36%	0.86%	0.09%	0.99%	0.37%	0.07%	0.06%	0.01%	0.00%	0.01%	0.05%	0.00%	0.00%
H4_24_35 K31su	2.27%	1.49%	0.83%	0.12%	0.02%	0.89%	1.74%	1.20%	3.61%	1.59%	1.03%	1.28%	0.87%	0.56%	1.16%	3.22%	1.92%
H4_24_35 K31cr	0.02%	0.00%	0.00%	0.01%	0.00%	0.00%	0.00%	0.08%	0.00%	0.00%	0.00%	0.01%	0.01%	0.01%	0.01%	0.05%	0.02%
H4_24_35 K31bu	0.10%	0.01%	0.01%	0.01%	0.02%	0.00%	0.00%	0.00%	0.00%	0.00%	0.03%	0.00%	0.00%	0.01%	0.00%	0.00%	0.00%
H4_24_35 K31pr	0.00%	0.00%	0.00%	0.21%	0.52%	0.02%	0.30%	0.00%	0.00%	0.01%	0.00%	0.00%	0.00%	0.00%	0.00%	0.10%	0.01%
GGVKR(H4_41_45)																	
H4_41_45 unmod	100%	100.00%	100.00%	43.23%	93.27%	100.00%	100.00%	100.00%	100.00%	100.00%	100.00%	100.00%	100.00%	100.00%	100.00%	100.00%	100.00%
H4_41_45 K44me1	0.00%	0.00%	0.00%	56.61%	0.00%	0.00%	0.00%	0.00%	0.00%	0.00%	0.00%	0.00%	0.00%	0.00%	0.00%	0.00%	0.00%
H4_41_45 K44me2	0.00%	0.00%	0.00%	0.00%	0.00%	0.00%	0.00%	0.00%	0.00%	0.00%	0.00%	0.00%	0.00%	0.00%	0.00%	0.00%	0.00%
H4_41_45 K44me3	0.00%	0.00%	0.00%	0.00%	0.00%	0.00%	0.00%	0.00%	0.00%	0.00%	0.00%	0.00%	0.00%	0.00%	0.00%	0.00%	0.00%
H4_41_45 K44ac	0.00%	0.00%	0.00%	0.00%	0.00%	0.00%	0.00%	0.00%	0.00%	0.00%	0.00%	0.00%	0.00%	0.00%	0.00%	0.00%	0.00%
H4_41_45 K44gl	0.00%	0.00%	0.00%	0.01%	0.02%	0.00%	0.00%	0.00%	0.00%	0.00%	0.00%	0.00%	0.00%	0.00%	0.00%	0.00%	0.00%
H4_41_45 K44hi	0.00%	0.00%	0.00%	0.00%	0.00%	0.00%	0.00%	0.00%	0.00%	0.00%	0.00%	0.00%	0.00%	0.00%	0.00%	0.00%	0.00%
H4_41_45 K44ma	0.00%	0.00%	0.00%	0.00%	0.00%	0.00%	0.00%	0.00%	0.00%	0.00%	0.00%	0.00%	0.00%	0.00%	0.00%	0.00%	0.00%
H4_41_45 K44su	0.00%	0.00%	0.00%	0.00%	0.02%	0.00%	0.00%	0.00%	0.00%	0.00%	0.00%	0.00%	0.00%	0.00%	0.00%	0.00%	0.00%
H4_41_45 K44cr	0.00%	0.00%	0.00%	0.00%	0.00%	0.00%	0.00%	0.00%	0.00%	0.00%	0.00%	0.00%	0.00%	0.00%	0.00%	0.00%	0.00%
H4_41_45 K44bu	0.00%	0.00%	0.00%	0.00%	0.00%	0.00%	0.00%	0.00%	0.00%	0.00%	0.00%	0.00%	0.00%	0.00%	0.00%	0.00%	0.00%
H4_41_45 K44pr	0.00%	0.00%	0.00%	0.15%	6.69%	0.00%	0.00%	0.00%	0.00%	0.00%	0.00%	0.00%	0.00%	0.00%	0.00%	0.00%	0.00%
GVLKVFLENVIR(H4_56_67)																	
H4_56_67 unmod	58.2%	90.94%	95.26%	84.74%	85.81%	61.66%	69.72%	84.55%	70.16%	88.48%	76.45%	85.49%	93.93%	66.96%	88.75%	83.10%	57.86%

H4_56_67 K59me1	14.0%	0.00%	0.01%	0.00%	0.00%	0.00%	0.00%	0.00%	0.00%	0.00%	0.00%	0.65%	0.00%	0.00%	0.00%	0.00%	0.27%
H4_56_67 K59me2	0.00%	0.00%	0.00%	0.20%	0.00%	0.00%	0.00%	0.00%	0.05%	0.00%	0.00%	0.00%	0.00%	0.00%	0.00%	0.00%	0.00%
H4_56_67 K59me3	14.9%	2.15%	1.42%	3.43%	5.12%	10.84%	4.83%	5.57%	10.29%	4.07%	2.44%	1.70%	1.77%	21.97%	2.41%	7.27%	4.55%
H4_56_67 K59ac	1.92%	0.57%	2.47%	10.72%	6.40%	19.66%	9.17%	5.54%	10.23%	0.00%	4.74%	4.29%	1.80%	2.45%	0.39%	0.92%	11.07%
H4_56_67 K59gl	3.39%	0.51%	0.23%	0.00%	0.00%	0.00%	3.60%	2.26%	0.68%	0.90%	1.39%	0.00%	0.08%	0.91%	2.01%	0.63%	14.03%
H4_56_67 K59hi	0.36%	0.79%	0.44%	0.00%	2.65%	2.79%	6.86%	1.08%	1.07%	0.73%	0.33%	2.09%	2.08%	0.65%	0.80%	0.54%	0.13%
H4_56_67 K59ma	2.90%	4.53%	0.17%	0.06%	0.00%	4.32%	4.33%	0.00%	2.77%	2.87%	12.91%	0.53%	0.09%	4.68%	2.04%	2.97%	1.64%
H4_56_67 K59su	1.50%	0.13%	0.00%	0.84%	0.00%	0.50%	1.46%	0.00%	1.76%	0.22%	1.65%	3.24%	0.05%	1.73%	2.30%	3.07%	7.52%
H4_56_67 K59cr	2.70%	0.38%	0.00%	0.00%	0.01%	0.12%	0.00%	0.78%	0.00%	2.51%	0.09%	1.92%	0.18%	0.56%	1.31%	0.69%	1.54%
H4_56_67 K59bu	0.00%	0.00%	0.00%	0.00%	0.01%	0.00%	0.00%	0.22%	2.97%	0.00%	0.00%	0.00%	0.00%	0.00%	0.00%	0.68%	1.37%
H4_56_67 K59pr	0.06%	0.00%	0.01%	0.00%	0.00%	0.10%	0.04%	0.00%	0.00%	0.22%	0.00%	0.08%	0.02%	0.08%	0.00%	0.13%	0.02%
DAVTYTEHAKR(H4_68_78)																	
H4_68_78 unmod	76.5%	74.59%	89.85%	95.93%	91.57%	90.12%	82.05%	84.09%	56.03%	89.27%	97.37%	80.69%	84.82%	90.73%	89.68%	86.13%	93.22%
H4_68_78 K77me1	10.0%	8.81%	0.03%	0.13%	0.34%	0.00%	0.01%	0.00%	0.00%	0.00%	0.01%	0.00%	5.88%	0.00%	0.00%	0.00%	1.22%
H4_68_78 K77me2	0.13%	0.01%	0.00%	0.01%	0.00%	0.00%	0.00%	0.02%	0.00%	0.01%	0.01%	0.00%	0.00%	0.00%	0.00%	0.00%	0.23%
H4_68_78 K77me3	0.01%	0.00%	0.07%	0.00%	0.01%	0.16%	0.00%	0.00%	0.03%	0.00%	0.07%	0.01%	0.00%	0.00%	0.05%	0.00%	0.00%
H4_68_78 K77ac	0.00%	0.00%	0.00%	0.00%	0.00%	0.00%	0.00%	0.00%	0.01%	0.00%	0.00%	0.00%	0.01%	0.00%	0.06%	0.00%	0.23%
H4_68_78 K77gl	0.00%	0.06%	0.19%	0.00%	0.01%	0.00%	0.00%	0.11%	0.00%	0.02%	0.04%	0.01%	0.00%	0.04%	0.06%	0.00%	0.07%
H4_68_78 K77hi	0.00%	0.42%	0.53%	0.00%	0.00%	0.47%	0.11%	0.10%	0.73%	0.10%	0.22%	0.12%	0.21%	0.30%	0.65%	1.10%	1.30%
H4_68_78 K77ma	0.00%	0.00%	0.01%	0.01%	0.00%	0.00%	0.00%	0.29%	0.00%	0.00%	0.02%	0.00%	0.00%	0.00%	0.00%	0.00%	0.11%
H4_68_78 K77su	0.08%	0.05%	0.03%	0.02%	0.00%	0.00%	0.00%	0.00%	0.07%	0.09%	0.03%	0.00%	0.00%	0.00%	0.09%	0.14%	0.02%
H4_68_78 K77cr	0.22%	0.48%	0.04%	3.71%	7.33%	0.00%	0.71%	0.18%	0.62%	0.16%	0.34%	0.43%	0.13%	0.14%	0.14%	0.52%	0.41%
H4_68_78 K77bu	12.6%	15.43%	9.24%	0.01%	0.42%	9.25%	16.49%	14.99%	42.18%	10.14%	1.59%	18.73%	8.90%	8.74%	9.23%	11.33%	2.48%
H4_68_78 K77pr	0.27%	0.16%	0.02%	0.17%	0.33%	0.00%	0.64%	0.22%	0.34%	0.21%	0.30%	0.01%	0.05%	0.04%	0.05%	0.77%	0.71%
KTVTAMDVVYALKR(H4_79_92)																	
H4_79_92 unmod	94.1%	67.49%	82.56%	88.42%	83.59%	98.91%	95.08%	46.58%	86.75%	84.48%	95.53%	58.23%	90.21%	86.12%	94.13%	72.44%	82.90%
H4_79_92 K91me1	0.00%	0.17%	0.00%	0.18%	1.23%	0.00%	0.00%	11.88%	0.00%	0.02%	0.00%	0.00%	0.00%	0.00%	0.00%	0.00%	0.00%
H4_79_92 K79me1	0.00%	0.35%	0.00%	0.24%	3.56%	0.00%	0.00%	39.39%	0.00%	0.03%	0.00%	0.00%	0.00%	0.00%	0.00%	0.00%	0.00%
H4_79_92 K79ac	0.52%	4.49%	3.06%	0.10%	0.37%	0.00%	0.00%	0.04%	0.35%	0.49%	0.00%	3.29%	0.00%	0.00%	0.00%	0.00%	0.00%
H4_79_92 K91ac	0.74%	6.19%	4.05%	0.15%	0.59%	0.00%	0.00%	0.14%	0.76%	2.11%	0.00%	5.96%	0.00%	0.00%	0.00%	0.00%	0.00%
H4_79_92 K79gl	0.13%	0.00%	0.40%	0.00%	0.06%	0.00%	0.00%	0.00%	0.00%	0.00%	0.00%	1.58%	0.00%	0.00%	0.00%	0.69%	0.38%
H4_79_92 K91gl	0.96%	0.00%	0.77%	0.00%	0.21%	0.00%	0.00%	0.00%	0.00%	0.00%	0.00%	2.54%	0.00%	0.00%	0.00%	1.11%	1.23%

H4_79_92 K79hi	0.75%	0.35%	0.13%	2.02%	1.99%	0.04%	1.13%	0.45%	3.65%	0.88%	1.54%	1.59%	0.52%	0.63%	0.38%	1.61%	0.59%
H4_79_92 K91hi	0.26%	0.67%	0.31%	4.48%	4.91%	0.22%	1.88%	0.14%	0.75%	1.99%	0.41%	3.56%	1.35%	2.30%	0.82%	0.65%	0.49%
H4_79_92 K79ma	0.13%	1.29%	1.59%	0.14%	0.07%	0.08%	0.06%	0.05%	0.06%	0.80%	0.13%	0.53%	0.05%	0.02%	0.00%	1.30%	0.35%
H4_79_92 K91ma	0.16%	1.87%	1.96%	0.35%	0.15%	0.12%	0.09%	0.09%	0.12%	1.71%	0.78%	0.85%	0.21%	0.02%	0.00%	6.09%	0.57%
H4_79_92 K79su	0.86%	4.12%	3.32%	0.93%	0.66%	0.05%	0.28%	0.33%	4.25%	3.45%	0.26%	12.44%	6.50%	6.97%	2.48%	3.65%	1.33%
H4_79_92 K91su	0.72%	1.20%	0.61%	2.92%	2.07%	0.13%	1.29%	0.16%	0.84%	0.79%	0.96%	0.78%	0.18%	0.71%	1.90%	0.95%	2.04%
H4_79_92 K79cr	0.02%	2.03%	0.00%	0.00%	0.00%	0.06%	0.00%	0.14%	0.13%	0.24%	0.09%	0.00%	0.10%	0.13%	0.00%	1.17%	0.08%
H4_79_92 K91cr	0.03%	2.54%	0.00%	0.00%	0.00%	0.09%	0.00%	0.23%	0.18%	0.52%	0.10%	0.00%	0.13%	0.14%	0.00%	1.93%	0.13%
H4_79_92 K79bu	0.00%	0.43%	0.00%	0.00%	0.00%	0.01%	0.04%	0.11%	0.14%	0.04%	0.00%	0.06%	0.07%	0.00%	0.03%	0.64%	0.24%
H4_79_92 K91bu	0.00%	0.70%	0.00%	0.00%	0.00%	0.02%	0.09%	0.26%	0.34%	0.11%	0.00%	0.17%	0.12%	0.00%	0.05%	0.97%	0.38%
H4_79_92 K79pr	0.20%	4.72%	0.47%	0.02%	0.15%	0.06%	0.02%	0.00%	0.49%	0.76%	0.05%	4.22%	0.28%	1.19%	0.05%	2.06%	3.09%
H4_79_92 K91pr	0.40%	1.39%	0.75%	0.04%	0.39%	0.19%	0.03%	0.00%	1.18%	1.58%	0.14%	4.21%	0.28%	1.77%	0.16%	4.74%	6.21%

Supplementary Table 4. ***In nucleo* acylation assay**. *In nucleo* assay in the presence of 1 µM and 10 µM of acyl-CoAs. Data represents the relative abundances of the modified peptides assuming the sum of the raw intensities of the unmodified and modified peptides as 100%. All results are shown as the average of 3 biological replicates.
